# A unique class of Zn^2+^-binding serine-based PBPs underlies cephalosporin resistance and sporogenesis in *Clostridioides difficile*

**DOI:** 10.1038/s41467-022-32086-6

**Published:** 2022-07-28

**Authors:** Michael D. Sacco, Shaohui Wang, Swamy R. Adapa, Xiujun Zhang, Eric M. Lewandowski, Maura V. Gongora, Dimitra Keramisanou, Zachary D. Atlas, Julia A. Townsend, Jean R. Gatdula, Ryan T. Morgan, Lauren R. Hammond, Michael T. Marty, Jun Wang, Prahathees J. Eswara, Ioannis Gelis, Rays H. Y. Jiang, Xingmin Sun, Yu Chen

**Affiliations:** 1 Department of Molecular Medicine, Morsani College of Medicine, University of South Florida, Tampa, FL 33612 USA; 2 Department of Global and Planetary Health, USF Genomics Program, Global Health and Infectious Disease Center, College of Public Health, University of South Florida, Tampa, FL 33620 USA; 3 Department of Chemistry, University of South Florida, Tampa, FL 33620 USA; 4 School of Geosciences, University of South Florida, Tampa, FL 33620 USA; 5 Department of Chemistry and Biochemistry, The University of Arizona, Tucson, AZ 85721 USA; 6 Department of Cell Biology, Microbiology, and Molecular Biology, University of South Florida, Tampa, FL 33620 USA; 7 Department of Medicinal Chemistry, Ernest Mario School of Pharmacy, Rutgers, the State University of New Jersey, Piscataway, NJ 08854 USA

**Keywords:** Antimicrobial resistance, X-ray crystallography, Bacteriology, Pathogens

## Abstract

Treatment with β-lactam antibiotics, particularly cephalosporins, is a major risk factor for *Clostridioides difficile* infection. These broad-spectrum antibiotics irreversibly inhibit penicillin-binding proteins (PBPs), which are serine-based enzymes that assemble the bacterial cell wall. However, *C. difficile* has four different PBPs (PBP1-3 and SpoVD) with various roles in growth and spore formation, and their specific links to β-lactam resistance in this pathogen are underexplored. Here, we show that PBP2 (known to be essential for vegetative growth) is the primary bactericidal target for β-lactams in *C. difficile*. PBP2 is insensitive to cephalosporin inhibition, and this appears to be the main basis for cephalosporin resistance in this organism. We determine crystal structures of *C. difficile* PBP2, alone and in complex with β-lactams, revealing unique features including ligand-induced conformational changes and an active site Zn^2+^-binding motif that influences β-lactam binding and protein stability. The Zn^2+^-binding motif is also present in *C. difficile* PBP3 and SpoVD (which are known to be essential for sporulation), as well as in other bacterial taxa including species living in extreme environments and the human gut. We speculate that this thiol-containing motif and its cognate Zn^2+^ might function as a redox sensor to regulate cell wall synthesis for survival in adverse or anaerobic environments.

## Abstract (web summary)

Antibiotics of the β-lactam class inhibit bacterial cell wall synthesis by targeting penicillin-binding proteins (PBPs). Here, Sacco et al. study the four PBPs present in the pathogen *C. difficile*, revealing unique structural features and shedding light on the mechanisms underlying β-lactam resistance in this organism.

## Introduction

C*lostridioides difficile* infection (CDI) is a condition where the anaerobic, spore-forming, bacterium *C. difficile* infects the large intestine, producing symptoms that range from mild-diarrhea to life-threatening colitis. As the most common hospital-acquired infection, the pathogenesis of CDI is well-understood^1,2^. Initially, antibiotics are administered for an unrelated infection or prophylaxis, causing the gut flora diversity to diminish. Without competition in the large intestine, *C. difficile* can easily proliferate, secreting toxins that cause cell death. The primary risk factor for CDI are broad-spectrum antibiotics, specifically those with weak activity against *C. difficile* and strong activity against other gut bacteria^3^. Currently, 3rd generation cephalosporins and clindamycin present the highest odds ratio for CDI (3.20, 2.86), followed by 2nd and 4th generation cephalosporins (2.23, 2.14)^4^. Other risk factors include treatment duration, age, overall health, and diet. Specifically, recent studies have found Zn^2+^-rich diets increase the likelihood and severity of CDI^5–7^.

Cephalosporins are members of the β-lactam family of antibiotics. These drugs achieve their bactericidal potency by irreversibly inhibiting penicillin-binding proteins (PBPs), particularly the transpeptidase (TPase) PBPs that cross-link cell wall peptidoglycan. Collectively, their enzymatic activity is essential and inactivating individual TPase PBPs can prove lethal to the organism^8,9^. Four annotated TPase PBPs exist in the genome of *C. difficile* R20291, a hypervirulent epidemic ribotype 027 strain: PBP1 (CDR20291_0712), PBP2 (CDR20291_0985), PBP3 (CDR20291_1067), and SpoVD (CDR20291_2544)^10,11^. PBP1 is the only bifunctional (class A) PBP that bears an additional glycosyltransferase (TGase) domain, which polymerizes the cell wall glycan chain^11,12^. In contrast, monofunctional TPases (class B PBPs) such as PBP2, PBP3, and SpoVD, must associate with a separate TGase, like those from the SEDS (shape elongation division sporulation) family, to polymerize the glycan units of peptidoglycan^13^. These four PBPs are highly conserved amongst major clades of *C. difficile* (>99% sequence identity, Supplementary Table 1), including reference strain 630, and those belonging to epidemic ribotypes 027, 078, and 106. In addition to these four strictly conserved PBPs, certain *C. difficile* strains possess a fifth TPase PBP with ~42% identity to *B. subtilis* PBP3. A recent study found PBP1 and PBP2 are essential for vegetative growth in R20291, while SpoVD and PBP3 are essential for sporogenesis^11^.

Cephalosporin resistance in *C. difficile* is well documented, but the underlying mechanism has, until this point, remained unclear^14^. Herein, we use a combination of experimental techniques to characterize the molecular basis of cephalosporin resistance in *C. difficile*. In doing so, we identify a unique role for Zn^2+^ in three of the four *C. difficile* TPase PBPs, which represent a new group of PBPs that have not been previously characterized.

## Results and discussion

### PBP2 is the main bactericidal target for β-lactam antibiotics but is poorly inhibited by cephalosporins

**Fig. 1:**
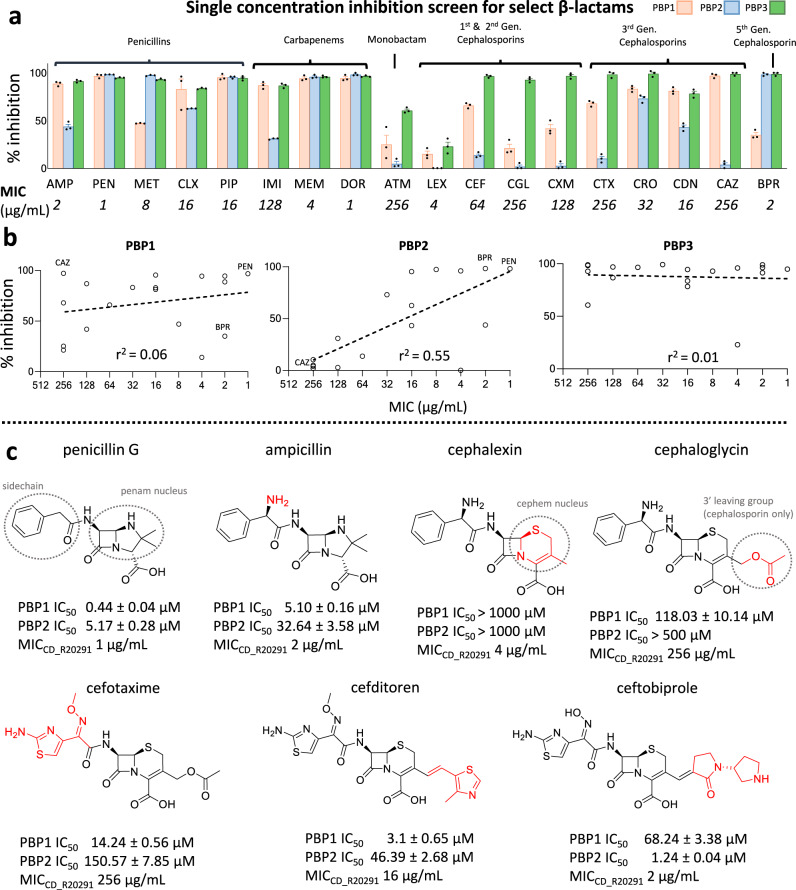
Biochemical and cellular inhibition profile of β-lactam antibiotics against major vegetative C. difficile TPase PBPs. **a** Select β-lactams were tested at 25 µM for their ability to compete with the binding of the fluorescent penicillin, bocillin. Their % inhibition are shown as a bar graph where each color represents inhibition against PBP1 (orange), PBP2 (blue), and PBP3 (green). Data are reported as the mean of three independent experiments ± SEM with errors bars representing SEM. Source data are provided as a Source Data file. The antibacterial inhibition (MIC) against C. difficile is listed directly below it (mean MIC reported from three biological replicates). As seen, the cephalosporins, which are poor inhibitors, also have low antibacterial potency. The relationship between antibacterial potency and biochemical inhibition is further illustrated in the scatterplot shown in panel **b**, where the log regression coefficient is listed. The high coefficient for PBP2 suggests it is the primary target of β-lactams. **c** IC_50_ values were subsequently determined for several β-lactams to demonstrate the chemical features that facilitate inhibition against PBP1 and PBP2. The sidechain, 3’ leaving group moieties, and the penam, and cephem nuclei are circled in the top row. Chemical change from the preceding drug (left to right) is colored in red. As shown, the cephem nucleus appears to be associated with significantly weaker inhibition against PBP1 and PBP2, but this can be overcome with chemical modifications to the sidechain and 3’ leaving group. AMP ampicillin, PEN penicillin G, MET methicillin, CLX cloxacillin, PIP piperacillin, IMI imipenem, MEM meropenem, DOR doripenem, ATM aztreonam, LEX cephalexin, CEF cephalothin, CGL cephaloglycin, CXM cefuroxime, CTX cefotaxime, CRO ceftriaxone, CDN cefditoren, CAZ ceftazidime, BPR ceftobiprole.

By comparing the antibacterial MIC of select members of each β-lactam class (penicillins, cephalosporins, carbapenems, and monobactams) we found resistance is more pronounced amongst cephalosporins (MICs > 64 µg/mL), consistent with previous reports^15^. Subsequent biochemical analysis of purified PBP1, PBP2, and PBP3 (Fig. 1a, Supplementary Fig. 1) demonstrate PBP1 and PBP2 are selectively insensitive to most cephalosporins, in contrast to the non-essential PBP3 which is potently inhibited by all β-lactams. Compared with PBP1 and PBP3, there was a significant correlation between PBP2 inhibition and MIC (*r*^2^ = 0.55). When cephalexin, which has no detectable inhibition against PBP1, PBP2, or PBP3, is omitted as an outlier, the *r*^2^ coefficient for PBP2 inhibition becomes *r*^2^ = 0.73 (Supplementary Fig. 2). Combined with previous genetic analysis^11^, these data suggest PBP2 is the main target through which most β-lactams achieve their bactericidal properties against *C. difficile* (Fig. 1b). This correlation is also evident between the MIC and PBP2 IC_50_ values for β-lactams that underwent further analysis (Fig. 1c, Supplementary Fig. 3).

While PBP1 is encoded by an essential gene, there was only a weak correlation between β-lactam inhibition and antibacterial activity (*r*^2^ = 0.06), just marginally higher than that of the non-essential PBP3 (*r*^2^ = 0.01). However, because PBP1 is a bifunctional PBP, gene-deletions fail to determine whether its TPase or TGase activity is essential. This experiment suggests the TGase domain, and not the TPase domain, probably represents the essential component of this enzyme. Notably, ceftazidime is a selective and potent inhibitor of PBP1, but has minimal impact on *C. difficile* growth (MIC = 256 µg/mL). Conversely, ceftobiprole was the most potent cephalosporin tested (MIC = 2 µg/mL) and is a selective and potent inhibitor of PBP2 with ~100% inhibition at 25 µM. The inhibition profile of these two cephalosporins independently support the hypothesis that PBP2 is the primary target for β-lactams, while the PBP1 TPase domain is likely less important for the action of β-lactams. This would suggest that the TGase domain of PBP1, rather than its TPase domain, is essential for vegetative growth.

### The cephem nucleus of cephalosporins is associated with weaker inhibition against PBP1 and PBP2

With few exceptions, cephalosporins poorly inhibited both PBP1 and PBP2, leading us to hypothesize that the cephem nucleus might confer weaker inhibition against these enzymes. We subsequently determined the IC_50_ values of seven β-lactam drugs, which would answer two outstanding questions: 1) is the cephem nucleus associated with weaker inhibition than those containing a penam nucleus and 2) if so, can chemical modifications to the 3’ leaving group and sidechain overcome intrinsic barriers to inhibition? A comparison of ampicillin with cephalexin, which is chemically identical except for the cephem nucleus, suggests the cephem nucleus is indeed responsible for the weaker inhibition towards PBP1 and PBP2 (Fig. 1c, Supplementary Fig. 4). Cephalexin, however, is atypical since it lacks a 3’ moiety that functions as a leaving group, which normally contributes to the energetics of cephalosporin binding^16^. By evaluating cephalosporins with different 3’ leaving groups (e.g., cephalogylcin) and side chains (e.g., cefotaxime), we demonstrate that modifications of these functional groups can improve inhibition against PBP1 and PBP2, and consequently, antibacterial potency (Fig. 1c). Among them, ceftobiprole was the best inhibitor of PBP2 (IC_50_ = 1.24 ± 0.04 µM) and has unique activity against Gram-positive bacteria expressing low-affinity PBPs, such as methicillin-resistant *Staphylococcus aureus* (MRSA), *Enterococcus faecalis*, *and Streptococcus pneumoniae*^17^. Notably, the 3’ vinyl group of ceftobiprole is a poor leaving group and remains adjoined after acylation. Strangely, cephalexin (MIC = 4 µg/mL) has similar antibacterial potency to ceftobiprole (MIC = 2 µg/mL) despite failing to inhibit any of the six TPases tested, including Ldt2 and Ldt3, two enzymes from the cysteine L,D transpeptidase family that also cross-link cell wall peptides^18–20^ (Supplementary Fig. 5), suggesting it may achieve bactericidal action by cumulative inhibition of non-essential PBPs, or another target altogether.

### Crystal structure of *C. difficile* PBP2 reveals a unique active site zinc-binding motif that contributes to protein stability and activity

**Fig. 2:**
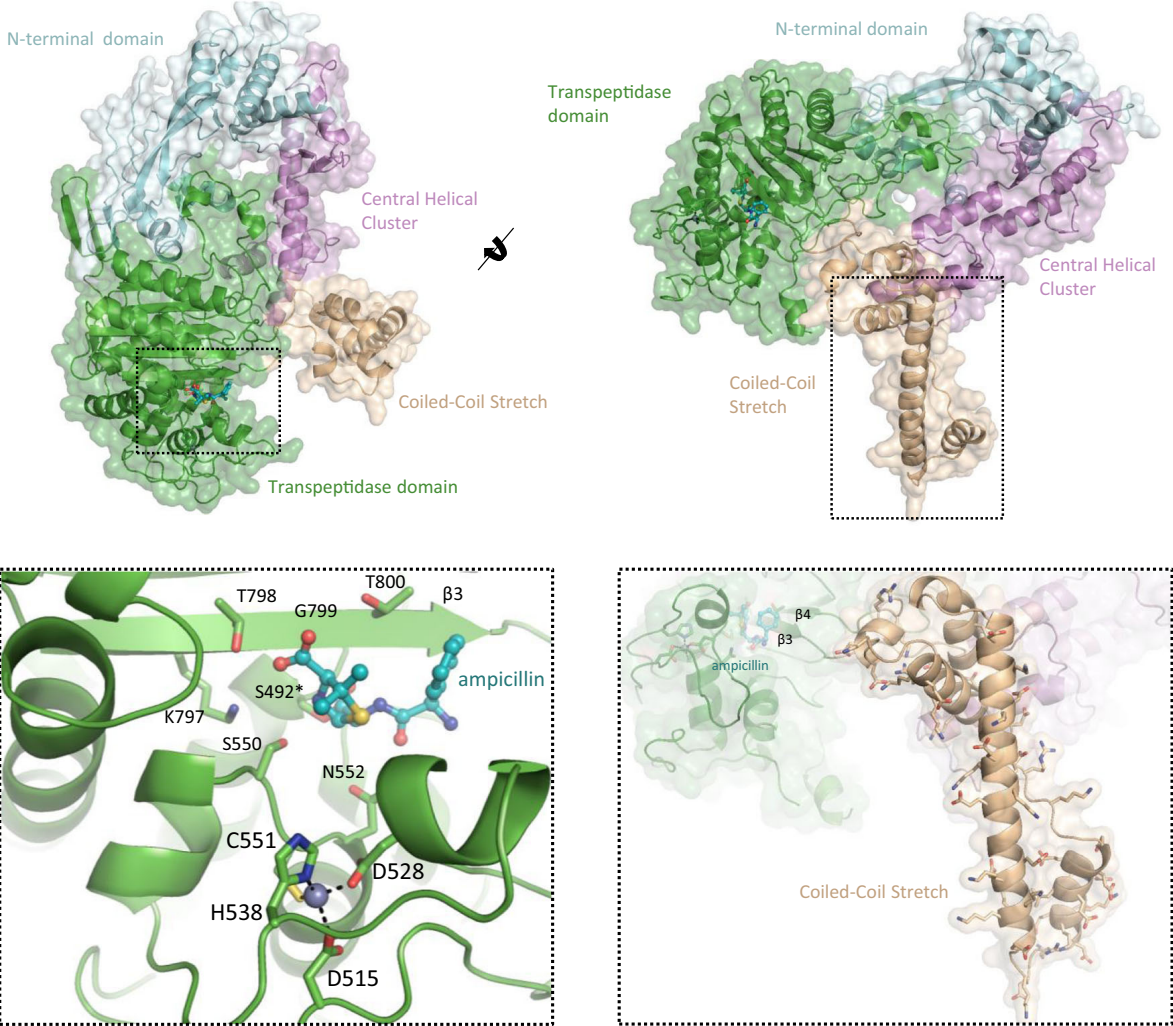
Crystal structure of the PBP2-ampicillin complex. The crystal structure of PBP2 in complex with ampicillin at 3.0 Å resolution reveals three novel structural features. This includes a novel Zn^2+^-binding motif in the active site (bottom left), a charged, elongated, helix connecting the β3-β4 strands of the TPase domain, termed the “coiled-coil stretch” (bottom right), and five roughly parallel α-helices, the “central helical cluster”.

*C. difficile* PBP2 is one of the largest known PBPs, with a MW of 111.14 kDa. As such, the primary sequence of PBP2 bears several contiguous regions with no known homology. For the first time, we present its crystal structure with ampicillin at 3.0 Å resolution (Fig. 2, Extended Data Supplementary Table 2). Besides being the first published structure of a *C. difficile* PBP, it is, to our knowledge, the largest PBP published to date (855 residues modeled: 91.37 kDa). The TPase domain adopts a canonical penicilloyl serine transferase fold, characterized by a five-stranded β-sheet that is sandwiched by three α-helices. The active site is found between the third, KTG-motif bearing strand of the five-stranded β-sheet (PBP numbering: β3 / overall: β15), an SXN motif across the channel, (S550-C551-N552) and two, nearly orthogonal α-helices that form the floor of the narrow substrate-binding cleft. One of these α-helices (α13) covalently binds to ampicillin through its catalytic S492. The second modeled region was the N-terminal domain (NTD, res. 1-110, 241-383), or “pedestal domain”, which has low sequence identity, but moderate structural homology to other TPase PBPs^9,21^. The architecture of the NTD is characterized by a “head” of three helices and an “anchor” formed by three β-strands.

PBP2 has two, long stretches of primary sequence with no known homology. The crystal structure shows these regions adopt two well-defined and distinct super-secondary structures that we have termed the “coiled-coil stretch” and the “central helical cluster”. The coiled-coil stretch (res. 805–915), is an elongated coiled-coil that lies orthogonally to the TPase domain (Fig. 2—bottom right). This domain is highly unique because it is found between the β3-β4 strands of the TPase domain, differing from other published PBPs which usually have an unstructured loop of ~5–20 residues at this position^9,21^. The linchpin of this assembly is an α-helix of 33 residues. Aliphatic residues are concentrated at helix-helix interfaces while a large number of charged residues are mostly in solvent exposed regions, the majority of which are positioned towards the bottom of this helix, suggesting it may interact with the outer leaflet of the membrane bilayer or a separate polar macromolecule. The “central helical cluster” is an insertion (res. 111–240) of five α-helices and two short β-strands connected by a β-turn between the 1st and 2nd α-helices of the NTD that lies between the coiled-coil stretch and the NTD. Extending throughout the core of PBP2, the central helical cluster forms contacts with all three subregions of the enzyme.

Perhaps the most notable feature of PBP2 is a highly unique Zn^2+^-binding motif adjoined to the active site (Fig. 2—bottom left). Normally concealed in the primary sequence, PBP2 represents the first known instance of an active site Zn^2+^-binding motif in a serine TPase PBP. The Zn^2+^ ion is coordinated by D515, D528, H538, and C551, where C551 is the X residue of the highly conserved SXN motif. The SXN motif plays an important role in substrate binding, suggesting the Zn^2+^ ion is important for maintaining the structural integrity and supporting the catalytic function of the active site^8,9^.

The discovery of a Zn^2+^ in the active site of PBP2, initially through X-ray crystallography (Fig. 2, Supplementary Fig. 6a), was subsequently confirmed with native-mass spectrometry (Supplementary Fig. 6b), quadrupole inductively coupled plasma mass spectrometry (Q-ICP-MS), as well as a fluorometric zinc quantification kit (Zn^2+^: protein = 0.91:1). Q-ICP-MS provides mass resolution to ~0.7 amu at a broad mass range and thus it can be utilized to unambiguously determine Zn^2+^ binding, by detecting and calibrating to various isotopes of Zn. To investigate its biochemical role, C551 and D515 were mutated to Ser and Asn. These mutations (Cys → Ser and Asp → Asn) were designed to displace the Zn^2+^ ion without disrupting structural integrity or generating artificial interactions within the active site. Indeed, D515N and C551S result in a 12-fold and 120-fold drop in ICP-MS intensity, respectively, indicating that the ability of the mutants to coordinate zinc becomes significantly compromised (Supplementary Fig. 6e). In addition, although WT PBP2 has a melting temperature (T_m_) of 64.45 ± 0.15 °C, C551S and D515N mutants demonstrate a bi-sigmoidal unfolding pattern with reduced T_m_, suggesting denaturation occurs sequentially and independently in two separate domains of the protein (C551S T_m1_ = 36.56 ± 0.29 °C, T_m2_ = 53.62 ± 0.13 °C; D515N T_m1_ = 35.10 ± 0.44 °C, T_m2_ = 56.01 ± 0.11 °C; Supplementary Fig. 6c). The altered melting temperature curves for the C551S and D515N mutants suggests this Zn^2+^-binding motif is critical for the structural stability of PBP2. Additionally, we find that both C551S and D515N have reduced affinity for the fluorescent penicillin, bocillin (PBP2_WT_ *K*_0.5_ = 2.95 ± 0.72 μM, PBP2_C551S_ *K*_0.5_ = 13.90 ± 1.84 μM, PBP2_D515N_ *K*_0.5_ = 16.72 ± 2.72 μM; Supplementary Fig. 6d), suggesting the loss of this Zn^2+^ indirectly influences catalytic activity.

### Comparison of unbound and complex PBP2 structures reveals ligand-induced conformational changes and narrow active site restricting cephem nucleus access

**Fig. 3:**
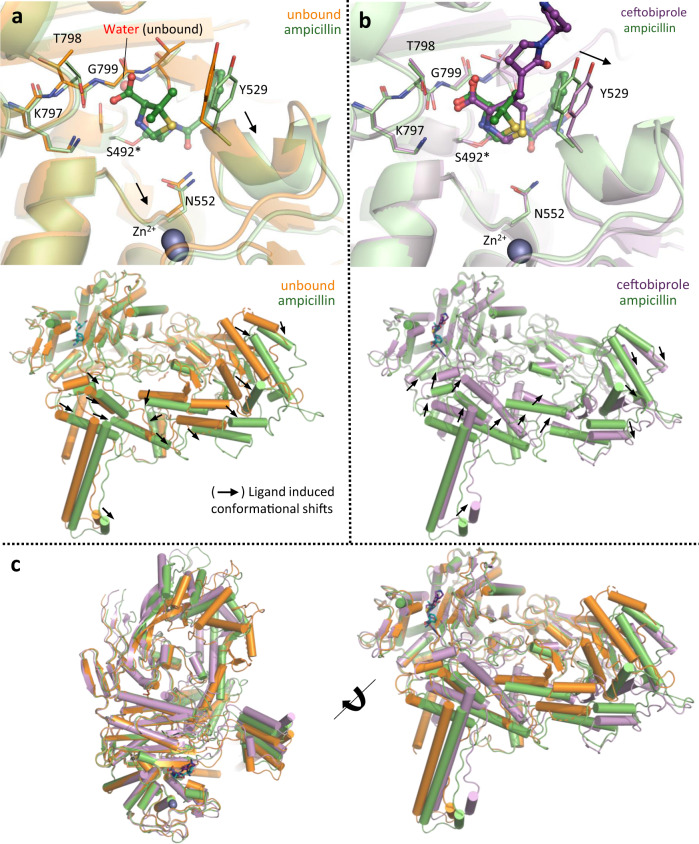
β-lactam binding opens the active site of PBP2 and induces global conformational changes. A superimposition of (**a**) unbound *C. difficile* PBP2 (orange) with ampicillin-bound form (green) shows β-lactam binding causes major conformational changes in the active site (top panel) and peripheral regions (bottom panel). Significant movements are indicated by arrows. **b** By comparing the complex structure of ampicillin (green) with ceftobiprole (purple) we find larger conformational changes in the active site (top panel) and overall (bottom panel) that are consistent with the larger size of the cephem core. **c** A superimposition of all three structures highlights the global conformation differences in all three structures, with movements mostly localized to the coiled-coil stretch, central-helix cluster, and NTD.

To delineate the structural basis for the cephalosporin insensitivity of PBP2, we solved structures of PBP2 in its unbound form (2.8 Å resolution; Fig. 3a) and with ceftobiprole (3.0 Å resolution; Fig. 3b). When unbound, the active site of PBP2 is occluded and K797, T798, and G799 of the highly conserved KTG motif are distorted. These distortions allow the catalytic serine to adopt a non-productive pose underneath the β3 strand, where it forms a hydrogen bond with a water molecule, making it inaccessible (Fig. 3a—top panel, Supplementary Fig. 7a, b). However, the active site opens upon ampicillin binding, causing substrate-recognition residues to translate 1–2 Å outwards and the KTG-bearing β3 sheet to shift downward, rigidify, and displace the water molecule. These movements expose the catalytic serine, freeing it for nucleophilic attack. The described active site rearrangements are mostly retained in the ceftobiprole complex (Fig. 3b—top panel, Supplementary Fig. 7c), but they are greater than those seen for ampicillin, such as the position of Y529 and adjacent residues, which is consistent with the larger size of the cephem nucleus (perimeter = 9.7 Å vs 8.5 Å in ampicillin; Fig. 3b). Consequently, the energetic barrier required for binding and acylation may be higher, and additional compensatory interactions must be formed to overcome this barrier. Thus, without multiple favorable interactions, like those of ceftobiprole, most cephalosporins have comparatively high IC_50_ values. While the active site conformational changes of PBP2 are significant, we observed even larger (>5–15 Å) rigid body translations outside of the TPase domain (Fig. 3a, b—bottom panel, Fig. 3c). These global conformational changes differ between the two complexes and involve regions that likely interact with essential accessory proteins, including the elongasome protein MreC and a SEDS-family transmembrane TGase MrdB (Supplementary Fig. 8a, b)^22,23^. Interestingly, the putative MreC binding cleft formed at the NTD “hinge” splays at different angles between the ampicillin and unbound structures, exposing a hydrophobic crevasse that is significantly narrower when bound to ampicillin (Supplementary Fig. 8c). Notably, in the ampicillin bound cleft there is electron density that may correspond to a glycerol that is not present in the unbound or ceftobiprole-bound structures. The unique conformations observed for all three structures of PBP2 suggest that occupation of the active site not only induces changes elsewhere in the enzyme, but the chemical identity of the ligand may actually contribute to these different poses. It is possible these movements may promote or negatively impact interactions with partner proteins depending on the nature of the occupying ligand, i.e., substrate (peptidoglycan peptide) or β-lactam. Meanwhile, interactions with protein partners may also influence ligand binding in the active site. Whether and how exactly these conformational changes contribute to regulation of PBP2 activity remains to be investigated. Taken together, the significant local and global conformational changes induced by ligand binding are highly unique for PBP2, even when compared with other β-lactam insensitive PBPs such as MRSA PBP2A^24,25^, providing the basis for activity regulation and intrinsic resistance to certain β-lactams.

### *C. difficile* and other anaerobic Firmicutes encode a novel family of PBPs with an active site Zn^2+^ ion

**Fig. 4:**
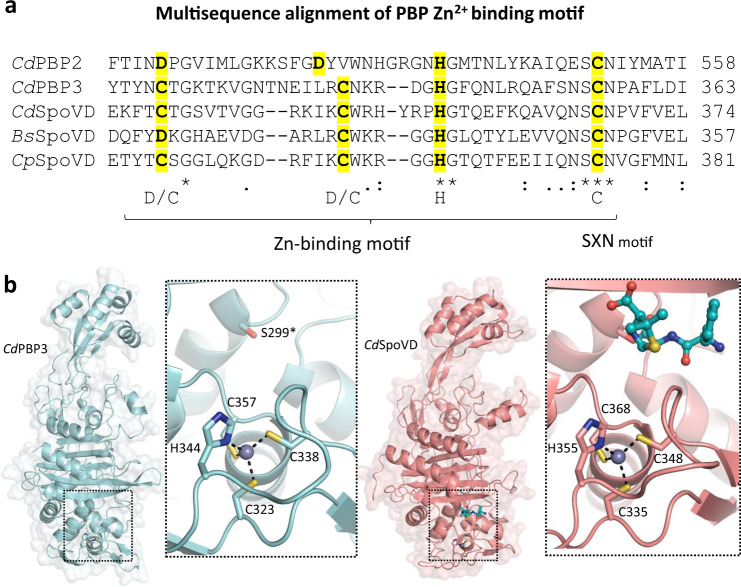
*C. difficile* and other spore-forming Firmicutes encode a unique family of Zn^2+^-binding PBPs. **a** Multiple sequence alignment uncovers a conserved Zn^2+^-coordinating tetrad in *C. difficile* PBP2, PBP3, SpoVD, and other spore-forming Firmicutes like *Bacillus subtilis* (Bs) and *Clostridium perfringens* (Cp) SpoVD. **b** The crystal structures of *C. difficile* PBP3 (cyan) and SpoVD (salmon) at 2.40 Å and 2.20 Å resolution reveal a shared fold and Zn^2+^-binding motif.

Though certain metallo-β-lactamases and carboxypeptidases rely on Zn^2+^ for catalysis, an active site Zn^2+^ has never been found in a serine TPase PBP prior to our characterization of *C. difficile* PBP2^26,27^. The discovery of this active site Zn^2+^ and subsequent decryption of the coordinating residues in the primary sequence led to the identification of other Zn^2+^-binding PBPs. Remarkably, in addition to *C. difficile* PBP2, we find this Zn^2+^-binding motif is also present in *C. difficile* PBP3 and SpoVD, meaning three of the four TPase PBPs, and all three class B PBPs in *C. difficile* have this motif. This motif was also found in SpoVD from several other model Firmicutes such as *Bacillus subtilis* and *Clostridium perfringens* (Fig. 4a). The presence of Zn^2+^ in *C. difficile* PBP3, *C. difficile* SpoVD, and *B. subtilis* SpoVD was subsequently confirmed by native-mass spectrometry as well as Q-ICP-MS (Supplementary Fig. 9). Like PBP2, the four residues in the Zn^2+^-binding motif of *C. difficile* PBP3 and SpoVD consist of an invariant His, a Cys from the X residue of the SXN motif, and two electron rich residues—in this case Cys instead of Asp. They also closely resemble the coordinating residues of the classical Cys_2_His_2_ zinc finger that binds to Zn^2+^ with high affinity^28^. The tight binding of Zn^2+^ in the *C. difficile* PBPs is supported by the observation that PBP2 activity was not affected by 2 mM EDTA and suggests that its presence in these proteins is unlikely an artifact from protein expression and purification in *Escherichia coli*.

Next, we determined the structures of PBP3 and SpoVD, at 2.40 Å and 2.20 Å resolution (Fig. 4b). Both SpoVD and PBP3 possess a canonical class B PBP fold that closely resembles *Pseudomonas aeruginosa* and *E. coli* PBP3^29,30^. Importantly, we find Zn^2+^ binds in an almost identical manner as PBP2 (Fig. 2), suggesting Asp and Cys are interchangeable at the first and second position. The significance of the Asp → Cys substitution between SpoVD/PBP3 and PBP2 is unclear. Whereas the redox-sensitive cysteine in the SXN motif for PBP2 can potentially function as a sensor for environmental oxygen levels, the replacement of Asp for Cys in both sporulation specific PBPs (PBP3 and SpoVD) may further enhance the sensitivity and/or subject them to additional regulation. Previous studies in *B. subtilis* SpoVD have found this motif, which was predicted to be a disulfide bond, interacts with the *B. subtilis* oxidoreductase StoA, suggesting it may function as a unique regulatory mechanism for PBP activity^31^.

### Prevalence of Zn^2+^-binding PBPs in anaerobes through convergent evolution suggests potential adaptation to counter oxygen toxicity

**Fig. 5:**
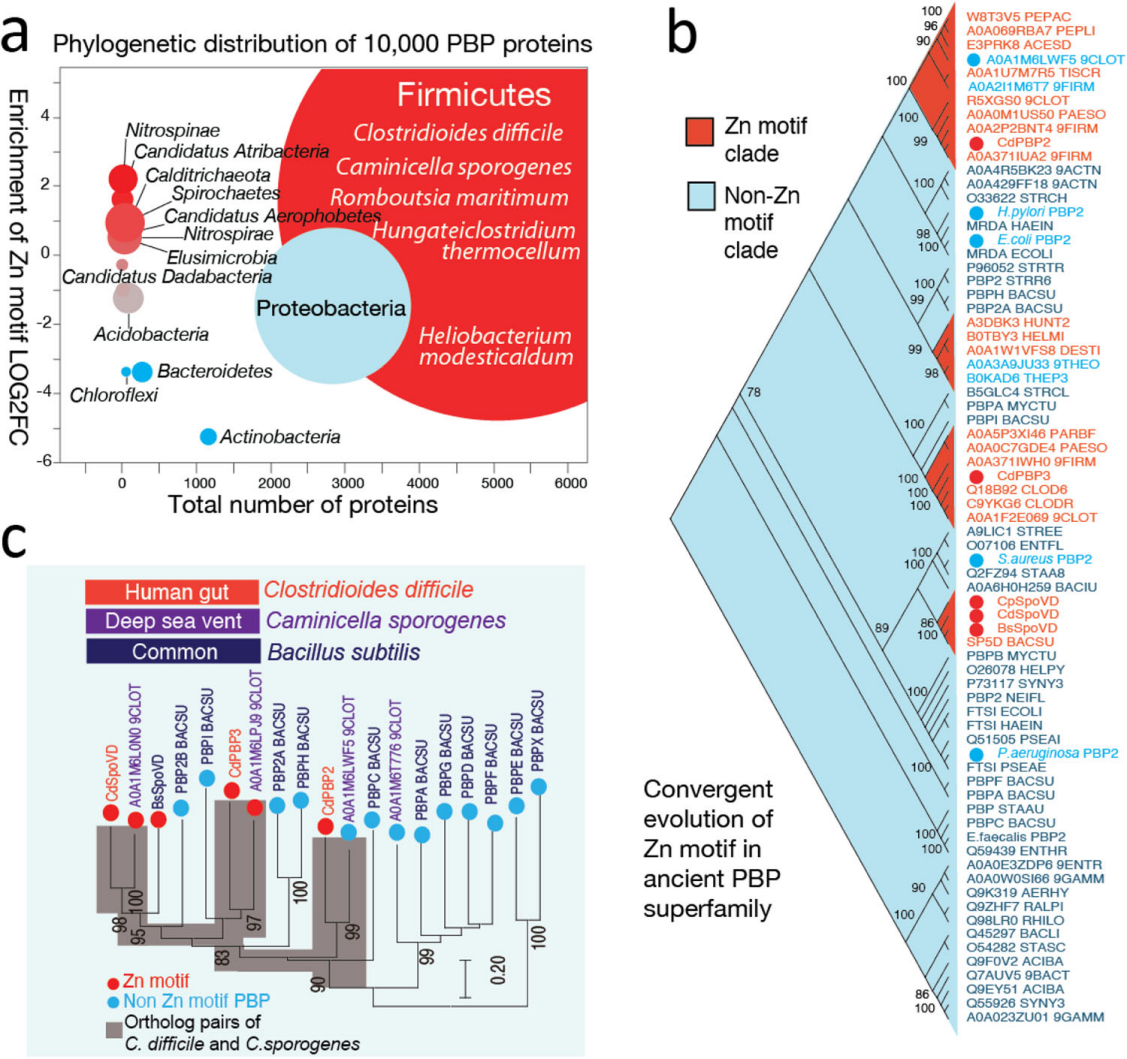
Phylogenetic distributions and evolutionary patterns of Zn^2+^-binding motifs in the PBP superfamily. Red indicates enrichment of Zn^2+^-binding PBP, blue indicates absence of Zn^2+^-binding PBPs. **a** Over 10,000 PBPs were analyzed and Zn^2+^-binding motifs were identified with a custom-built hidden Markov model (HMM). Circle size represents the number of species. Representative species are listed for Firmicutes. **b** Structurally diverse PBP superfamily members from diverse taxa were used to build a maximum likelihood tree, revealing Zn^2+^-binding PBPs emerged through convergent evolution in the ancient PBP superfamily. These PBPs have evolved multiple times from independent clades. Bootstrap values are listed next to the branches. **c** *C. difficile* and the deep-sea vent Gram-negative bacterium *C. sporogenes* share the same set of PBP proteins, two of which (SpoVD and PBP3 homologs) bear Zn^2+^-binding motifs.

To investigate the presence of Zn^2+^-binding PBPs in a wider range of bacterial species, a hidden Markov model (HMM) based on the immediate flanking sequences of the Zn^2+^-binding motif was used to examine the UniProt database (www.uniprot.org). A total of over 5000 PBPs were retrieved containing the Zn^2+^-binding motif, and a control set of over 5,000 PBPs without the Zn^2+^-binding motif was used for taxa distribution analysis (Supplementary Data 1). The vast majority of bacteria bearing this motif belong to the Firmicutes phylum and most are anaerobes (Fig. 5a). Representative species include those from extreme environments such as deep-sea vents (*Caminicella sporogenes*), oil fields (*Hungateiclostridium thermocellum*), volcanic ashes and hot springs (*Heliobacterium modesticaldum*)^32–34^, suggesting Zn^2+^-binding PBPs might confer survival advantages in adverse conditions, especially anoxic ones. Next, a set of phylogenetically diverse PBPs based on the PFAM seed PBP domain collections (Transpeptidase; PF00905) were selected to examine the evolutionary pattern of Zn^2+^-binding PBPs (Supplementary Data 2). Unexpectedly, we find the Zn^2+^-binding motif independently evolved through convergent evolution from the ancient PBP superfamily, as shown by the four clades in the phylogenetic tree (Fig. 5b). Interestingly, in the PBP2 clade, there are two proteins without a Zn^2+^-binding motif, suggesting it can be gained or lost in a relatively short evolutionary span. For example, *Caminicella sporogenes*, an anaerobic, Gram-negative bacterium isolated from a deep-sea vent, shares a similar set of PBPs (PBP2, PBP3, and SpoVD) with *C. difficile* (Fig. 5c). Although *C. sporogenes* PBP3 and SpoVD possess a Zn^2+^-binding motif, its PBP2 orthologue (Uniprot A0A1M6LWF5_9CLOT) does not, despite its high sequence identity/similarity score (41.2%/60.3%), as defined by the BLOSUM62 matrix, and predicted structural, similarity to *C. difficile* PBP2 (Supplementary Fig. 10). This degree of homology suggests *C. sporogenes* PBP2 can acquire the capacity for Zn^2+^-binding through one or two mutations. Furthermore, because *C. difficile* PBP2 is implicated in vegetative growth and PBP3/SpoVD facilitate sporulation, the Zn^2+^-binding motif may be required for different biological processes, depending on the specific bacterium and the natural habitat.

Notably, bacteria containing Zn^2+^-binding PBPs within the PBP2 clade are exclusively anaerobic. Considering that cysteine residues, including those involved in Zn^2+^-binding, are involved in redox sensing in other proteins^35,36^, we hypothesize that the Zn^2+^-binding PBPs may function as a regulatory mechanism that can shut down cell wall synthesis or promote sporulation when confronted with molecular oxygen and/or reactive oxygen species, in conjunction with the previously studied transcription-level responses^37^. This hypothesis is partly supported by the aforementioned observation that *B. subtilis* SpoVD is regulated by the oxidoreductase StoA^31^. Furthermore, as many antibiotics achieve at least part of their bactericidal effects through reactive oxygen species^38^, such mechanisms may possibly contribute to antibiotic resistance in bacteria such as *C. difficile*.

### All classes of β-lactam antibiotics and the Zn^2+^-chelators calprotectin and TPEN inhibit *C. difficile* sporulation

**Fig. 6:**
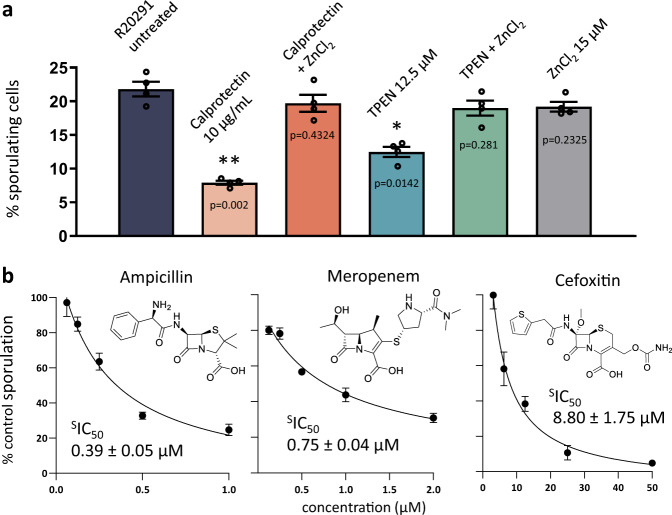
Zn^2+^-chelating agents TPEN, calprotectin, and all classes of β-lactams disrupt C. difficile sporulation. **a** Anti-sporulation effects of the chemical chelator TPEN and the biological chelator calprotectin, an innate immune system protein. This inhibition can be reversed through restoring growth media with Zn^2+^. Four biologically repeats were performed. Data between two groups were analyzed by student’s unpaired *t*-test for statistical significance. Data are expressed as mean ± SEM. Differences were considered statistically significant if *p* < 0.05 (*) vs R20291, *p* < 0.01 (**) vs R20291, and nonsignificant where undesignated. **b** All three major classes of β-lactams- ampicillin (penicillins), meropenem (carbapenems), and cefoxitin (cephalosporins) were assessed at sub-MIC concentrations (½ MIC), revealing these antibiotics are potent inhibitors of sporulation (indicated by sporulation IC_50_, or ^S^IC_50_) via inhibition of PBPs required for spore cortex assembly, such as PBP3 and SpoVD. Three independent experiments were performed for each concentration, and each data point is presented as mean ± SEM with error bars representing SEM. Source data are provided as a Source Data file.

Several lines of evidence suggest dietary Zn^2+^ increases the severity and likelihood of CDI^5,39^, but the pleiotropic roles of Zn^2+^ have obfuscated the exact mechanism by which it facilitates pathogenesis. Recent studies have found that calprotectin, a protein secreted by the immune system in response to intestinal inflammation, inhibits *C. difficile* growth^6^. Interestingly, calprotectin is a chelator of divalent cations such as Ca^2+^ and Zn^2+^. Using calprotectin and the hexadentate ligand TPEN, we show sporulation is significantly inhibited at 10 µg/mL and 12.5 µM, respectively (Fig. 6a). This anti-sporulation effect is reversed by supplementing the media with Zn^2+^, suggesting it is specific to Zn^2+^, even though most certainly not limited to PBP activity. The consistent presence of a Zn^2+^ motif in all three class B *C. difficile* PBPs (PBP2, PBP3, and SpoVD), and its apparent importance for enzymatic activity and sporulation, suggests Zn^2+^ may be uniquely associated with growth of the vegetative cell and spore-cortex. Furthermore, these PBPs may have evolved to take advantage of the anaerobic environment of the colon, which is rich in excreted trace metals such as Zn^2+^^40^^,^. Thus, while Zn^2+^ likely plays multiple important roles in *C. difficile* growth, it at least impacts sporulation and may contribute to the relationship between CDI and colonic Zn^2+^ concentration. Other anti-sporulating agents include cephamycins, a class of cephalosporins that inhibit the enzymatic activity of SpoVD^41^. Because PBPs are integral for spore-cortex formation, we hypothesized that, in addition to cephamycins, all β-lactam antibiotics should also reduce sporulation. Here we show that ampicillin (sporulation IC_50_, or ^s^IC_50,_ 0.39 ± 0.05 µM) and meropenem (^s^IC_50_ 0.75 ± 0.04 µM) are more efficacious than cefoxitin (^s^IC_50_ 8.80 ± 1.75 µM) in vitro (Fig. 6b). Although the use of β-lactams to inhibit *C. difficile* sporulation is not practical, they may be uniquely effective against other pathogens such as *C. perfringens* and *Bacillus anthracis*. Nonetheless, SpoVD and PBP3 are very promising drug targets. A selective, non-bactericidal inhibitor of these enzymes could be paired with the standard treatment regimen, thus mitigating the likelihood of recurrent infection and transmission associated with spore formation.

#### Concluding remark**s**

Independently, PBPs and CDI are heavily studied subjects, yet very little research exists for the PBPs of *C. difficile*. This study helps delineate the molecular basis for cephalosporin resistance in *C. difficile* and the high risk-factors associated with these antibiotics. More specifically, we demonstrate that *C. difficile* PBP1 and PBP2 are poorly inhibited by most cephalosporins, resulting in lower antibacterial activity. The insensitivity of *C. difficile* to cephalosporins, coupled with broad-spectrum activity against gut bacteria, contribute to the extraordinarily high odds-ratio associated with this class of antibiotics. Our discovery of a new group of Zn^2+^-binding PBPs offers further insights into the Zn^2+^-dependence of *C. difficile*, while identifying a novel motif that is present in a wide range of bacteria. Collectively, the characterization of these PBPs leads to new and important questions about the role and regulation of these PBPs, their novel domains, and the Zn^2+^-binding motif as it relates to vegetative cell growth, division, and sporogenesis in this unique pathogen. The evaluation of their inhibition profiles, relative essentiality, and crystal structures also provides a structural framework for future antibiotic discovery against *C. difficile*, especially considering the recent headway of novel non-β-lactam chemical scaffolds as PBP inhibitors^42^.

## Methods

### Protein cloning and purification

The coding sequence corresponding to the TPase domain (376–765) of PBP1 (CDR20291_0712), the soluble constructs of PBP3 (CDR20291_1067), Ldt2 (CDR20291_2601), Ldt3 (CDR20291_2843), PBP2 (CDR20291_0985), SpoVD (CDR20291_2544), and the crystallization constructs of PBP2 (CDR20291_0985) and PBP3 (CDR20291_1067), were amplified from *C. difficile* R20291 genomic DNA^10^. The soluble sequence of BSU_15170 (BsSpoVD) was amplified from the genomic DNA of *B. subtilis* strain 168^43^. The expression vector was modified from pETGST, in which the thrombin cleavage site was replaced by the TEV (named pETGSTTEV) or ULP1 (named pETGSTSUMO) cleavage site. The vector was digested and the PCR fragments for each protein was inserted into the multi-clone site accordingly. PBP1 (376–765) PCR fragment was obtained by primers 5’GGTCGCTAGCTCTTCATCTGTAGAAGACCTTATCGAAAGTG and 5’GGTCAAAAGCTTTTAATTTTTAGGAACTTCAAATTCAGTAACCGTTAA and inserted into NheI/HindIII site; PBP2 36–962 fragment (5’GGTCGCTAGCTATGAATATTACAATGAGTTAGCTGAGAATAAAAC and 5’GGTCAACTCGAGTTATAATCCCATATAAGTTCCAATAACTTCTC) and Ldt2 fragment (5’GGTCGCTAGCAAA CATGTGATTATAGTAAATTCAAGAAAAAATAC and 5’GGTCAACTCGAGTTATTTTGCAAGAATATCCAT TAATTTATCCATTAC) were inserted into NheI/XhoI sites; Ldt3 fragment (5’GGTCAACATATGAGAGGATGG ATATGTGTGAAATTAACTAAAC and 5’GGTCAACTCGAGTTAATGAATTATAACTGTTGTTGTATCTGG) and PBP3 42-554 fragment (5’GGTCAACATATGGGTGAT AAATACAAACAAAGTGTTGAATCA and 5’GGTCAACTCGAGTTATTTTATACTTTCACATATTTCCTTAAA TATAGG) were inserted into the NdeI/XhoI site. SpovD 38-583 fragment (5’GGTCGCTAGCGGAAATTGGTTGAGTACA AAAGCACTAGAAC and 5’GGTCAACTCGAGTTATGGTTTAACTCCCAAATATTTTAAAGAGTC) was inserted into the NheI/XhoI site of the pETGSTSUMO vector. PBP2 D515N and C551S mutants were generated using the QuikChange Lightning Site-Directed Mutagenesis Kit (Agilent Technologies). Cloned vector was then transformed into BL21(DE3) pLysS *E. coli*. Standard overnight cultures were grown in LB media containing chloramphenicol and kanamycin and used to inoculate 1 L LB cultures. To investigate the identity of the metal ion in the PBP2 active site, M9 minimal media supplemented with 15 µM ZnSO_4_ or CuCl_2_ were used to prepare two special versions of PBP2 protein. Cultures were grown until the optical density (OD_600_) reached ~0.7. Protein expression was then induced with 0.5 mM isopropyl-β-d-thiogalactopyranoside (IPTG) overnight at 20 °C. Cells were harvested via centrifugation at 4000 × *g* for 10 min at 4 °C. The cell pellet was suspended with a solution of 20 mM Tris pH 8.0, 200 mM NaCl, 20 mM imidazole, and one dissolved Thermo Scientific™ Pierce Protease Inhibitor Tablet. Cells were lysed with a sonicator using a 10 s sonication/15 s rest cycle for 15 min. Cell lysate was centrifuged at 45,000 × *g* for 35 min and supernatant was loaded onto a HisTrap HP affinity column (GE Healthcare). A linear gradient of buffer B (20 mM Tris pH 8.0, 300 mM NaCl, 500 mM imidazole, and 10% glycerol) was applied to elute the recombinant protein, which usually occurred at 30% buffer B. These fractions were pooled, and buffer exchanged three times into protease buffer (20 mM Tris pH 8.0, 10% glycerol) using an Amicon Ultra centrifugal filter (Sigma-Aldrich). Purified His6-tagged TEV protease was added to all proteins at a 1:20 ratio (except for SpoVD) and incubated overnight at 4 °C. Pure His6-tagged ULP1 protease was added to SpoVD at 1:20 ratio and incubated overnight at 4 °C. The digested protein was re-loaded onto the HisTrap HP affinity column. Flowthrough was collected, concentrated, and loaded to a HiLoad 16/60 Superdex 75 size exclusion column (GE Healthcare) where it ran at a flow rate of 0.5 mL/min in 20 mM Tris pH 8.0, 150 mM NaCl. Peak fractions were combined, and purity assessed (>95%) with gel-electrophoresis. The identity of each enzyme was subsequently characterized with gel-electrophoresis, native-mass spectrometry, bocillin or nitrocefin binding, and/or X-ray crystallography.

### X-ray crystallography

8 mg/mL PBP2 was incubated with 1 mM ampicillin for 2 h at room temperature. Crystals were grown by mixing 1.5 µL of the protein + inhibitor solution with 1.5 µL of well solution: 15% PEG 4000, 0.2 M ammonium sulfate, and 0.1 M sodium citrate pH 5.6. Cover slips containing the drops were sealed and equilibrated over 1 mL of well solution for one week at 20 °C, producing many plate-like crystals. These crystals were then crushed into seeds. This method was repeated, but instead of mixing the well stock with protein, diluted seed stock was used to control the rate of nucleation, yielding diffraction quality crystals. Crystals were then briefly soaked in a cryoprotectant solution of 30% PEG 4000, 0.2 M ammonium sulfate, 0.1 M sodium acetate pH 5.6, 15% glycerol and flash frozen in liquid nitrogen.

Surprisingly, PBP3 readily crystallized in the same conditions as PBP2 (15% PEG 4000, 0.2 M ammonium sulfate, 0.1 M sodium citrate pH 5.6). However, they were highly sensitive to glycerol in the cryoprotectant solution. To address this, crystals were crushed into seeds and diluted into the crystallization solution supplemented with 5% glycerol. Crystals were regrown by mixing 12 mg/mL protein with the 5% glycerol diluted seed stock. After reaching full size in a week, 2 µL of a cryoprotectant solution containing 25% PEG 4000 and 30% glycerol was added directly to the drops for ~20 s and crystals were flash-frozen in liquid nitrogen.

### Model building and refinement

X-ray diffraction data was collected on the Structural Biology Center (SBC) 19-ID and Southeast Regional Collaborative Access Team (SER-CAT) 22-ID beamlines at the Advanced Photon Source in Argonne, IL and processed and scaled with the CCP4 versions of iMosflm and Aimless^44–46^. Resolution was cut off when the following criteria were met: overall R_merge_ ≤ 10%, outer shell i/σi ≥ 2, and outer shell completeness ≥ 80%. Initial models were obtained using the MoRDa package of the online CCP4 suite^47^. Models were then processed with PHENIX Autobuild^48,49^. Unsolved regions were manually traced with a polyalanine backbone using COOT and refined with Refmac5^50,51^. Initially, aided with the secondary-structure prediction software PSIPRED^52^, α-helices were generated by tracing the electron density map, and bulky, aromatic sidechains such as tryptophan were fit into their corresponding positions. This allowed us to solve the sequence of adjacent residues. Iterations were generated until individual sidechains could be resolved. Two flexible regions with ambiguous electron density were left unmodelled: 357-370 of the NTD and 624–692 of the TPase domain. This region in the TPase domain likely contains three parallel α-helices, but we were unable to confidently model the density. Likewise, a short segment of 197–216 in the NTD was left unmodelled for PBP3. All structural images were generated using PyMol (Schrödinger, LLC)^53^. Topology diagrams were generated with the assistance of Pro-origami^54^.

### Zinc quantification assay

Zinc quantification assays were performed using a Fluorometric Zinc Quantification Kit (Abcam). Protein was denatured with equal volume of 7% TCA solution and centrifuged at 13,000 × *g* for 5 min. After protein denaturation, the supernatants were neutralized using 1 M Na_2_CO_3_ with 1/10 the sample volume. Samples were vortexed and kept on ice for 5 min before being used for reaction. A 100 µM ZnCl_2_ standard solution was prepared to perform 1:2 serial dilutions to generate a standard curve. 50 µL of ZnCl_2_ standards, prepared protein samples, and blank buffer control were added to a 96-well black polystyrene optical bottom plate (Thermo Fisher Scientific). PBP2 was tested at 2-fold, 5-fold, and 10-fold dilutions as well as PBP1 as a negative control, and NDM-1 as a positive control. PBP2 C551S was tested at 2-fold, 5-fold, and 10-fold dilutions to test for effect of mutation on Zn^2+^ binding. 50 µL of Zn Detection Assay buffer was added to each well and incubated for 10 min. Fluorescence intensity was measured at 485/525 nm (Ex/Em) at RT using a BioTek Cytation 5 plate reader (BioTek Instruments).

### Bocillin inhibition assay

Reactions were performed in a 96-well polystyrene plate with a final volume of 60 μL at 37 °C in 1X Tris-buffered saline (20 mM Tris, 150 mM NaCl). For IC_50_ values, inhibitor was serially diluted with a two-fold scheme so the highest concentration had a final value of 1 mM or 500 µM, and the final concentration was either 0.97 µM or 0.49 µM. Single concentration screens were performed at 25 µM. Protein was then diluted and added to the well for a final concentration of 1 µM and incubated for 15 min at 37 °C. 1 µL of bocillin (BOCILLIN™ FL Penicillin, Sodium Salt, Thermo Fisher Scientific) was added to each well for a final concentration of 20 µM, and incubated for 10 min at 37 °C. Reactions were killed with 10 µL of 6X SDS loading buffer and pooled or directly loaded onto a 15-well Novex Wedgewell 8% Tris-Glycine Gel (Thermo Fisher Scientific) and ran at 175 V for ~1 h. Gels were then imaged with a Gel Doc XR + imager (Bio-Rad Laboratories) for 6 s with the fluorescein blot filter setting and analyzed using ImageJ^55^. All values were normalized to background intensity and divided by the average of three control intensities (no inhibitor) for % inhibition value. Assays were performed in duplicate or triplicate and IC_50_ values were calculated using Graphpad Prism with a 4-parameter logistic fit. Unprocessed gels for Supplementary Figs. 1 and 6d are provided in the source data.

### Nitrocefin inhibition assay

Reactions were performed in a 96-well polystyrene plate with a final volume of 60 μL at 37 °C in 20 mM Tris pH 7, 200 mM NaCl. Inhibitors were diluted for a final concentration of 25 µM. Protein was then added to the well for a final concentration of 1 µM and incubated for 15 min at 37 °C. 1 µL of nitrocefin (Millipore Sigma) was then added to each well for a final concentration of 20 µM, and reaction progress was monitored using a BioTek Cytation 5 plate reader (BioTek Instruments) at 488 nm and 37 °C for 1 h. Reaction rates were normalized to the control (no inhibitor) for % inhibition values. Assays were performed in duplicate and mean % inhibition values was calculated.

### Thermal shift assay

Thermal shift assays were performed using differential scanning fluorometry. Purified protein was diluted in TBS and combined with SYPRO™ Orange (Invitrogen) for a final protein concentration of 3 µM and final SYPRO™ Orange concentration of 1X, with a final volume of 20 µL. Fluorescence was measured using The Applied Biosystems® 7900HT Fast Real-Time PCR (Thermo Fisher Scientific) from 25 °C to 99 °C with a ramp step of 0.3 °C. Melting temperature values were determined by fitting sigmoidal fluorescence intensity curves with a 4-parameter logistic fit.

### Minimum inhibitory concentration (MIC) determination

The MICs of antibiotics on *C. difficile* strain R20291 were determined as described previously^56^. Antibiotics were added to wells of 96-well microplates containing R20291 cultures at a density of 0.5 McFarland (100 µL per well) in BHI medium to make final concentrations of antibiotics ranging from 128 µg/mL to 0.5 µg/mL at a two-fold dilution. The plates were incubated at 37 °C for 24 h, and MIC was determined as the lowest concentration that completely inhibits the bacterial growth in the wells. MIC determination was repeated three times.

### Sporulation assays

Sporulation assays were performed as described previously with minor modifications^57–59^. Briefly, *C. difficile* R20291 cells were cultured to mid-log phase in BHIS medium supplemented with 0.1% taurocholate (Sigma) at 37 °C in an anaerobic chamber. A mixture of 70:30 sporulation medium (70% SMC medium and 30% BHIS medium containing 63 g Bacto peptone, 3.5 g protease peptone, 11.1 g BHI medium, 1.5 g yeast extract, 1.06 g Tris base, 0.7 g ammonium sulfate, and 15 g agar per liter) was prepared. Cultures were subsequently diluted to an optical density of 0.5 at OD_600_, then 150 µl of R20291 cultures in the presence/absence of calprotectin at 10 μg/ml or TPEN at 12.5 μM or ZnCl_2_ at 15 μM were spread over the surface of a 70:30 medium agar plate. Approximately 24 h after the start of stationary phase (T_24_), *C. difficile* cells were scraped from the surface of the plate with a sterile inoculating loop and suspended in ~5 mL BHIS to an OD_600_ = ~1.0. To assess ethanol-resistant spore formation, 500 µL of samples from the sporulation medium were removed from the anaerobic chamber and mixed 1:1 with 95% ethanol for 15 min to kill vegetative cells. The samples were then returned to the anaerobic chamber, 100 µl of the ethanol-treated cultures were mixed with 100 µl of 10% taurocholate, and the mixtures were plated onto BHIS agar to induce *C. difficile* spore germination. The ethanol-resistant CFU/mL was determined after incubation for 24 h and was divided by the total CFU/mL of the non-ethanol-treated cultures. Four biological replicates were performed.

### PBP sequence analysis

To examine the common protein domain families for discovery of patterns of site-specific evolutionary conservation related to Zn^2+^-binding PBPs, profile hidden Markov models (profile HMMs) were built for probabilistic domain searches^60^. HMMER (v.3.3.2)^61^ uses ensemble algorithms to consider all possible sequences alignments, each weighted by likelihood of positive scores. The sequences used as an input are listed in Supplementary Data 2. The databases of UniProtKB and SwissProt were used as targets for the search. For phylogenetic analysis, a set of most representative ‘seeds’ sequences were selected from the superfamily of over 200,000 members, the phylogenetically diverse domains of DD-transpeptidase (PF00905) were used analyze the evolutionary patterns.

### Native mass spectrometry

All proteins were buffer exchanged into 0.2 M ammonium acetate using Bio-Spin P-6 Size Exclusion Spin Columns (BioRad) as previously described^62^. Native MS was performed using a Q-Exactive HF quadrupole mass spectrometer with Ultra-High Mass Range research modifications (Thermo Fisher Scientific). All proteins were ionized using nano-electrospray ionization in positive mode using 1.1–1.5 kV of spray voltage at 200 °C. Samples were analyzed with a 2000–15,000 *m/z* range and the resolution was set to 15,000. The trapping gas was set to 5 for all samples and 50 V was applied in the source to aid in desolvation. Data were deconvolved and analyzed using UniDec^63^.

### Inductively coupled plasma mass spectrometry

A series of dilutions of PBP2 or PBP3 expressed in either rich (LB) or minimal media supplemented with ZnSO_4_−7H_2_O were prepared in 20 mM Tris, pH = 8.0, 200 mM NaCl to cover a concentration range of 0.2−8.25 μM. These dilutions were run on at the USF Center for Geochemical Analysis on a Perkin Elmer Nexion 2000P Quadrupole ICP-MS in He collision mode to minimize NaAr interference caused by the NaCl buffer and by sulfate at masses 63, 64, and 66 respectively. Conditions on the ICP-MS were as follows: RF power was fixed at 1600 W with nebulizer flow at 1.02 mL/min. Auxiliary and plasma gas flows were set to 1.2 and 15 mL/min respectively and pump speed was set to 18 rpm which delivers ~0.3 mL/min of sample to the spray chamber. The rejection parameter for the interference was set at 0.45. This setting significantly reduces count rates, but also minimizes the blank resulting in an enhanced signal to noise ratio. The lowest standard was 0.8 μg/L which was between 3 and 10 times over background for ^65^Cu and ^64^Zn, ^66^Zn, and ^68^Zn. We chose to reject the data for ^63^Cu as concentrations were still anomalously high for samples prepared in the buffer solution likely due to very high Na in the buffer and in turn NaAr interference at mass 63.

### Reporting summary

Further information on research design is available in the Nature Research Reporting Summary linked to this article.

## Supplementary information

Supplementary Information

Reporting Summary

Description of Additional Supplementary Files

Supplementary Dataset 1

Supplementary Dataset 2

## Data Availability

Source data are provided with this paper. All crystal structures have been deposited in the RCSB Protein Data Bank (PDB) with accession IDs of: *C. difficile* PBP2 + ampicillin (PDB ID 7RCW), unbound PBP2 (PDB ID 7RCX), PBP2 + ceftobiprole (PDB ID 7RCY), SpoVD + ampicillin (PDB ID 7RCZ), and unbound PBP3 (PDB ID 7RD0). Raw X-ray data is available upon request. All protein sequence data is freely accessible at https://www.uniprot.org/ and https://www.ncbi.nlm.nih.gov/ Source data are provided with this paper.

## Source data

Source data
