# Supplementary material for: A unique class of Zn2+-binding serine-based PBPs underlies cephalosporin resistance and sporogenesis in Clostridioides difficile
Source: Nat Commun. 2022 Jul 28;13:4370. doi: 10.1038/s41467-022-32086-6 (PMC9334274; doi:10.1038/s41467-022-32086-6)
Supplement: Supplementary file 1 — Supplementary Information [file 41467_2022_32086_MOESM1_ESM.pdf]

**Supplementary Data for**

# A unique class of $Zn^{2+}$-binding serine-based PBPs underlies cephalosporin resistance and sporogenesis in *Clostridioides difficile*

Michael D. Sacco[1], Shaohui Wang[1], Swamy R. Adapa[2], Xiujun Zhang[1], Eric M. Lewandowski[1], Maura V. Gongora[1], Dimitra Keramisanou[3], Zachary D. Atlas[4], Julia A. Townsend[5], Jean R. Gatdula[1], Ryan T. Morgan[1], Lauren R. Hammond[6], Michael T. Marty[5], Jun Wang[7], Prahathees J. Eswara[6], Ioannis Gelis[3], Rays H.Y. Jiang[2], Xingmin Sun[1*], and Yu Chen[1*]

[1]Department of Molecular Medicine, Morsani College of Medicine, University of South Florida, Tampa, FL, 33612, United States

[2]Department of Global and Planetary Health, USF Genomics Program, Global Health and Infectious Disease Center, College of Public Health, University of South Florida, Tampa, FL, 33620, United States

[3]Department of Chemistry, University of South Florida, Tampa, FL, 33620, United States

[4]School of Geoscience, University of South Florida, Tampa, FL, 33620, United States

[5]Department of Chemistry and Biochemistry, The University of Arizona, Tucson, AZ, 85721, United States

[6]Department of Cell Biology, Microbiology, and Molecular Biology, University of South Florida, Tampa, FL, 33620, United States

[7]Department of Medicinal Chemistry, Ernest Mario School of Pharmacy, Rutgers, the State University of New Jersey, Piscataway, NJ, 08854, United States

*Corresponding Authors

Yu Chen, Tel: 813-974-7809, email: ychen1@usf.edu
Xingmin Sun, Tel: 913-974-4553, email: sun5@usf.edu

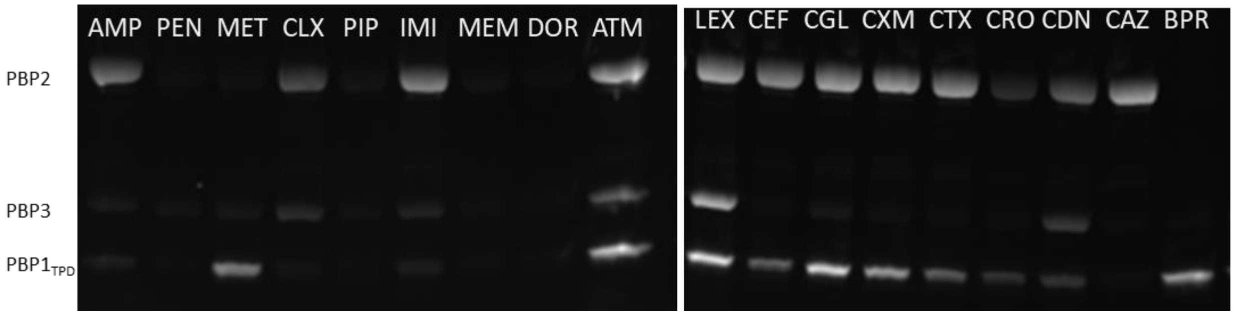


**Supplementary Figure 1| Single concentration inhibitor screen of different β-lactams against *C. difficile* PBP1 (transpeptidase domain only), PBP2, and PBP3.** Each β-lactam was assessed at 25 µM for its ability to compete with the binding of bocillin, a fluorescent penicillin. Thus, the fluorescence of each band is inversely proportional to inhibition. This experiment was performed three times independently. % inhibition was calculated as the mean of these three experiments and this value, with SEM was reported in Fig. 1a. Source data are provided as a Source Data file. AMP – ampicillin; PEN – penicillin G; MET – methicillin; CLX – cloxacillin; PIP - piperacillin; IMI – imipenem; MEM – meropenem; DOR – doripenem; ATM – aztreonam; LEX – cephalexin; CEF – cephalothin; CGL – cephaloglycin; CXM – cefuroxime; CTX – cefotaxime; CRO – ceftriaxone; CDN – cefditoren; CAZ – ceftazidime; BPR – ceftobiprole, CPT-- ceftaroline

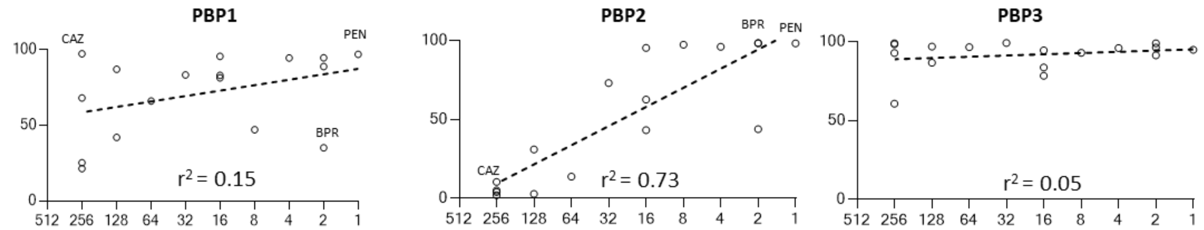


**Supplementary Figure 2 | Relationship between antibacterial potency (MIC) and % biochemical inhibition with cephalexin omitted**. Scatter plot showing MIC vs % inhibition at 25 μM (Fig. 1, Supplementary Fig. 1). Here, cephalexin was removed as an outlier. Cephalexin is highly unique amongst the tested β-lactams because it has little to no biochemical inhibition against any of the tested PBPs, yet has moderate antibacterial potency against *C. difficile* growth (MIC = 4 μg/ mL). The log correlation coefficient is labelled at the bottom of each graph.

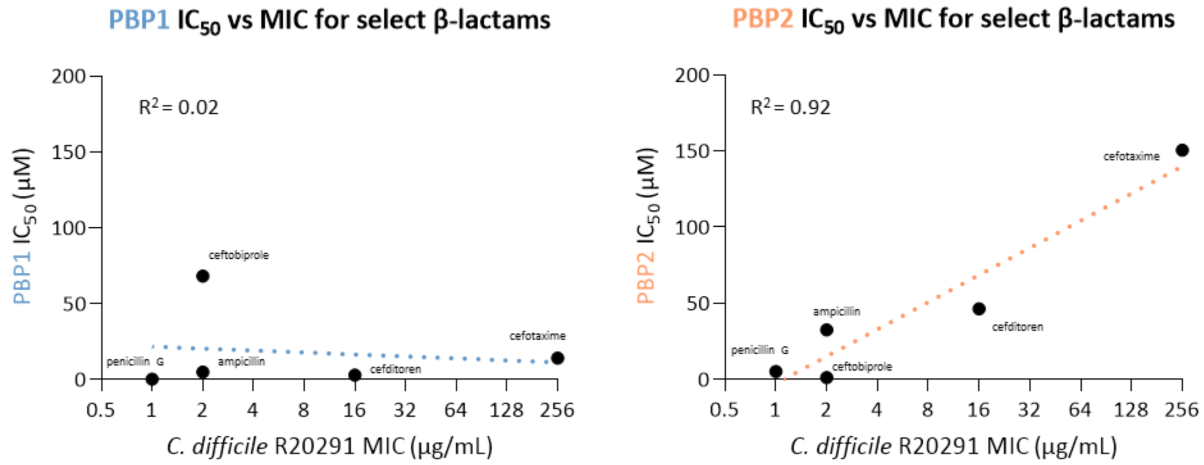


**Supplementary Figure 3 | Relationship between $IC_{50}$ and antibacterial potency for select β-lactams against *C. difficile*.** Log correlation between $IC_{50}$ and MIC against *C. difficile* R20291 with residual plot shown as dashed line. Cephalexin and cephaloglycin were excluded because $IC_{50}$ could not be accurately determined, especially against PBP2.

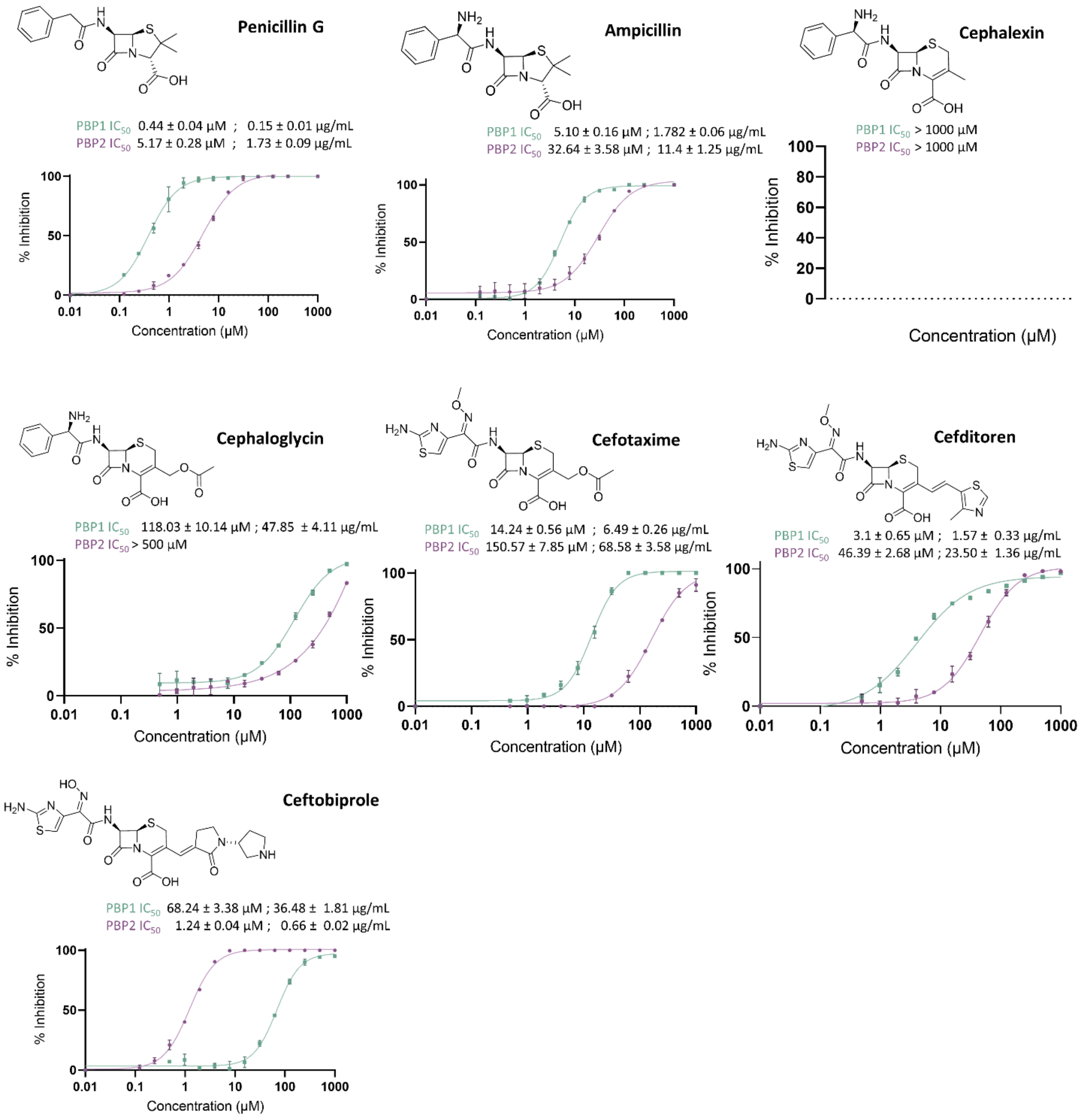


**Supplementary Figure 4| Inhibition curves for select β-lactams against *C. difficile* PBP1 and PBP2.** $IC_{50}$ values were determined from a non-linear four-parameter logistic fit and are reported in μM and μg/mL with SEM. For each dataset n=2. Source data are provided as a Source Data file.

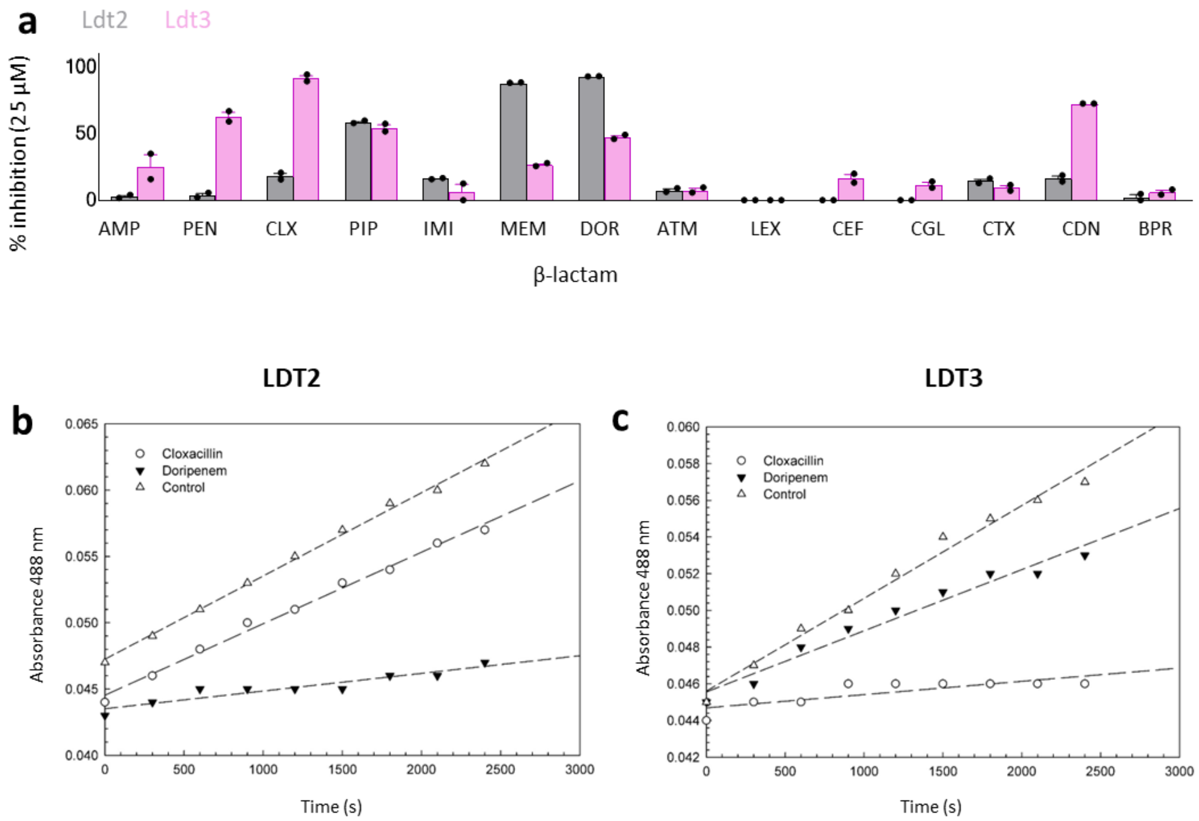


**Supplementary Figure 5 | Inhibition of Ldt2/Ldt3 nitrocefin hydrolysis by different β-lactam antibiotics at 25 µM** % inhibition are the mean of two experiments performed independently with error bars shown as SEM. Source data are provided as a Source Data file. Representative reaction curves of the nitrocefin hydrolysis assay are shown in the bottom panels. β-lactams were weaker inhibitors of Ldt2 and Ldt3 compared to the tested PBPs. Meropenem, doripenem, and piperacillin were the best inhibitors of Ldt2, while cloxacillin and cefditoren were the best inhibitors of Ldt3. AMP – ampicillin; PEN – penicillin G; CLX – cloxacillin; PIP -piperacillin; IMI – imipenem; MEM – meropenem; DOR – doripenem; ATM – aztreonam; LEX – cephalexin; CEF – cephalothin; CGL – cephaloglycin; CTX – cefotaxime; CDN – cefditoren; BPR – ceftobiprole.

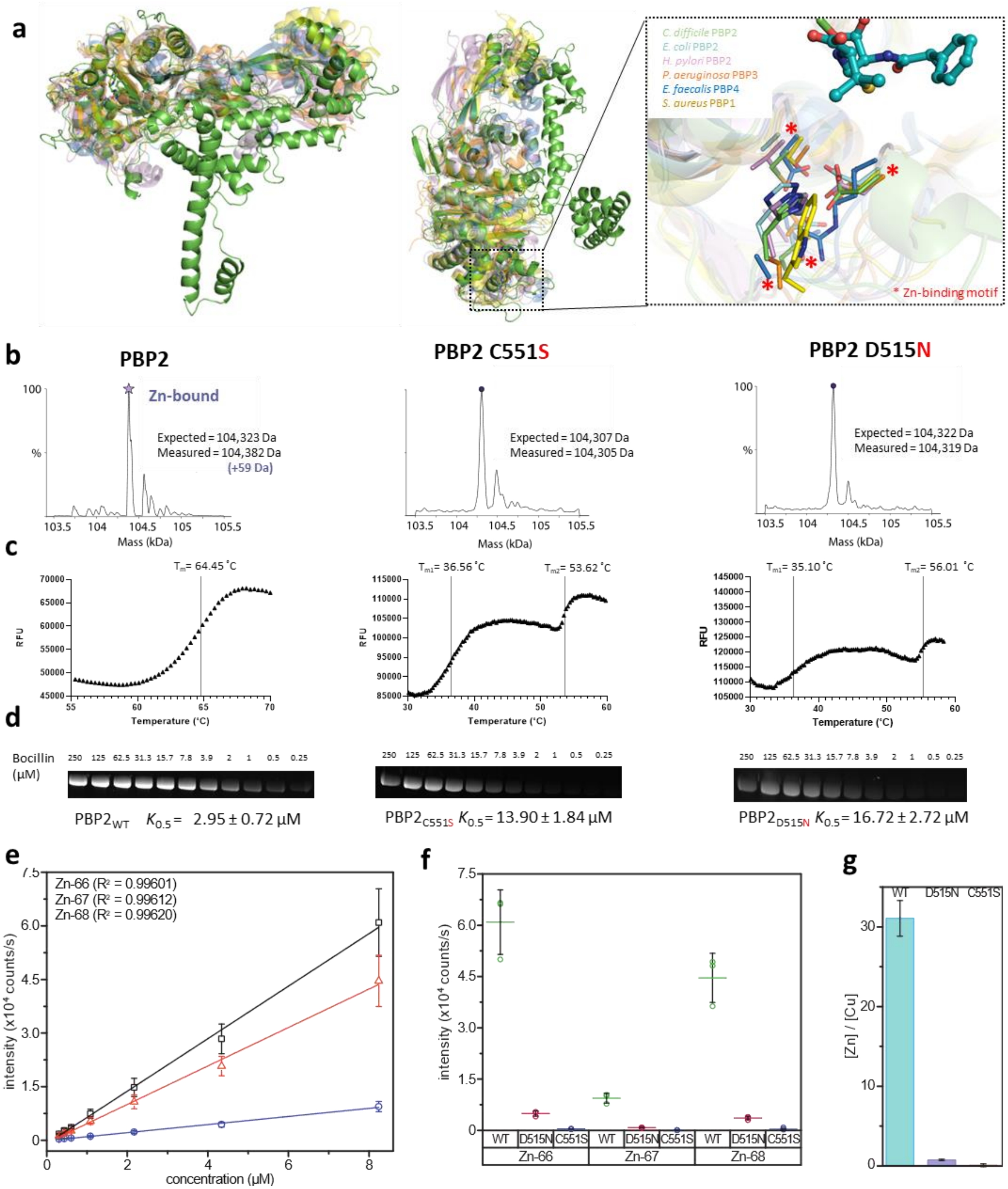

**Supplementary Figure 6| A unique $Zn^{2+}$-binding motif influences ligand binding and protein stability in PBP2. (a)** Structural superimposition of PBP2 with related homologues from select bacteria (left). The residues forming the $Zn^{2+}$ -binding motif from PBP2 are divergent amongst PBPs (right). **(b)** Native mass spectrometry, **(c)** melting temperature assay, and **(d)** bocillin titration for PBP2, PBP2 C551S, and PBP2 C551N. Data are the mean $K_{0.5}$ of two separate experiments fit using a 1-site binding nonlinear regression fit with standard deviation reported.Note, the raw data shown is one representative of the triplicates performed. **(e)** ICP-

MS response for the three stable Zn isotopes (expressed as counts/s) exhibit a strong correlation to PBP2 concentration. For each concentration, error bars represent standard deviations of six technical measurement repeats. **(f)** The zinc coordination defective mutants D515N and C551S cause a dramatic drop in ICP-MS response. Data recorded at 8.25 µM for wild-type (WT), D515N and C551S PBP2 is shown as dot plots, highlighting the three technical values (circles), the mean (horizontal lines) and the SD (error bars). **(g)** The ratio of zinc over copper concentration for WT-, D515N- and C551S-PBP2 indicate high selectivity of the metal binding site for zinc over copper for the WT enzyme. Cu-63 was excluded to avoid NaAr interference at mass 63. Metal concentration ratios are calculated at 8.25 µM PBP2. Error bars represent propagated SD of the ratios. Source data are provided as a Source Data file.

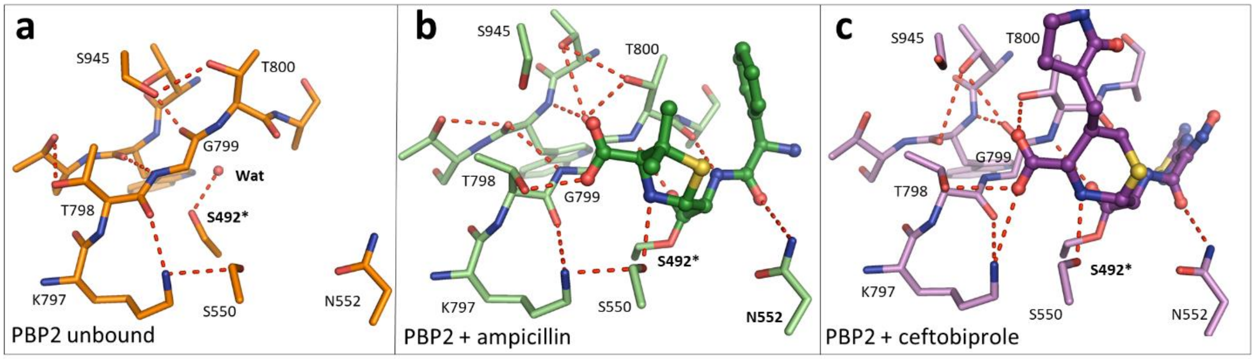


**Supplementary Figure 7| Residues involved in ligand binding demonstrate structural rearrangement upon binding.** The catalytic core of PBP2 **(a)** unbound, **(b)** with ampicillin, and **(c)** with ceftobiprole. Ligand binding induces conformational changes involving key residues, including the KTG motif, Asn552, and the catalytic Ser492*. These movements are accompanied by distinct hydrogen bonding patterns. Shown as red dashes, these hydrogen bonds were calculated using the FindHBond plugin of UCSF Chimera[1]

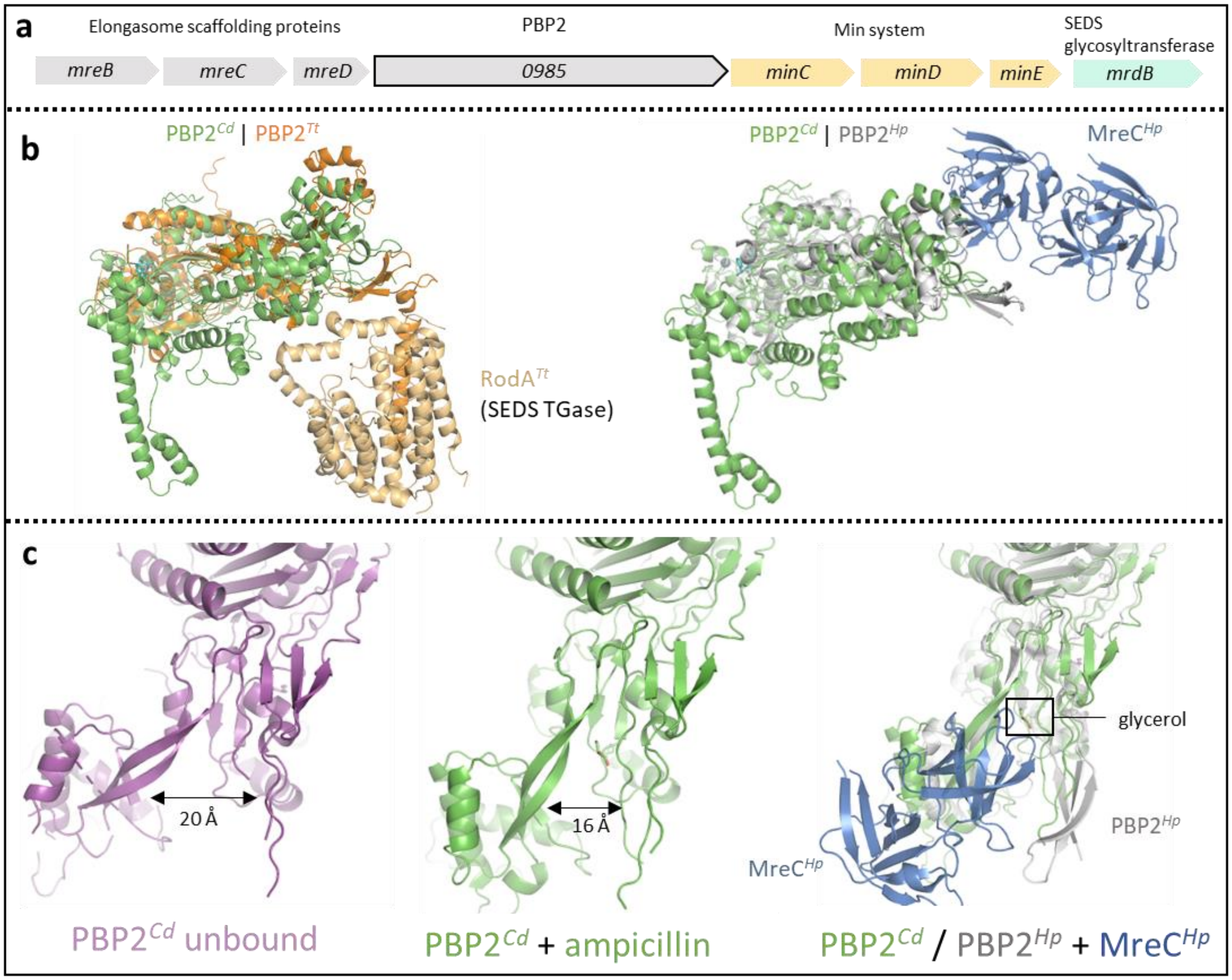


**Supplementary Figure 8| Projected assembly for the peptidoglycan synthase of the elongasome. (a)** The gene encoding PBP2 is in a locus with several genes that are essential for cell elongation and division. PBP2 is the gene product of CDR20291_0985, the terminal gene of the mreBCD operon (*mreB*, *mreC*, *mreD*; grey). CDR20291_0985 is also adjacent to the genes encoding the Min system (*minC*, *minD*, *minE*; yellow), which facilitate the placement of the septal Z-ring. Because PBP2 is a monofunctional class B transpeptidase, it must rely on *mrdB* (teal), a transmembrane SEDS glycosyltransferase four genes downstream, to polymerize the sugar units of peptidoglycan. **(b)** Sideview of *C. difficile* PBP2 superimposed with *Thermus thermophilus* PBP2 *T. thermophilus* PBP2 + RodA (left, PDB 6PL5), and with *Helicobacter pylori* PBP2 + MreC (right, PDB 5LP5) **(c)** The NTD of unbound (purple) and ampicillin-bound (green) PBP2 are splayed at different angles, resulting in a larger exposed crevasse for the unbound-form. This region is putatively involved in the binding of MreC, based on the complex structure of *H. pylori* PBP2 and MreC (right). In the ampicillin complex, there is electron density corresponding to a glycerol molecule where a portion of MreC (modelled from *H. pylori* MreC PDB 5LP5) normally binds.

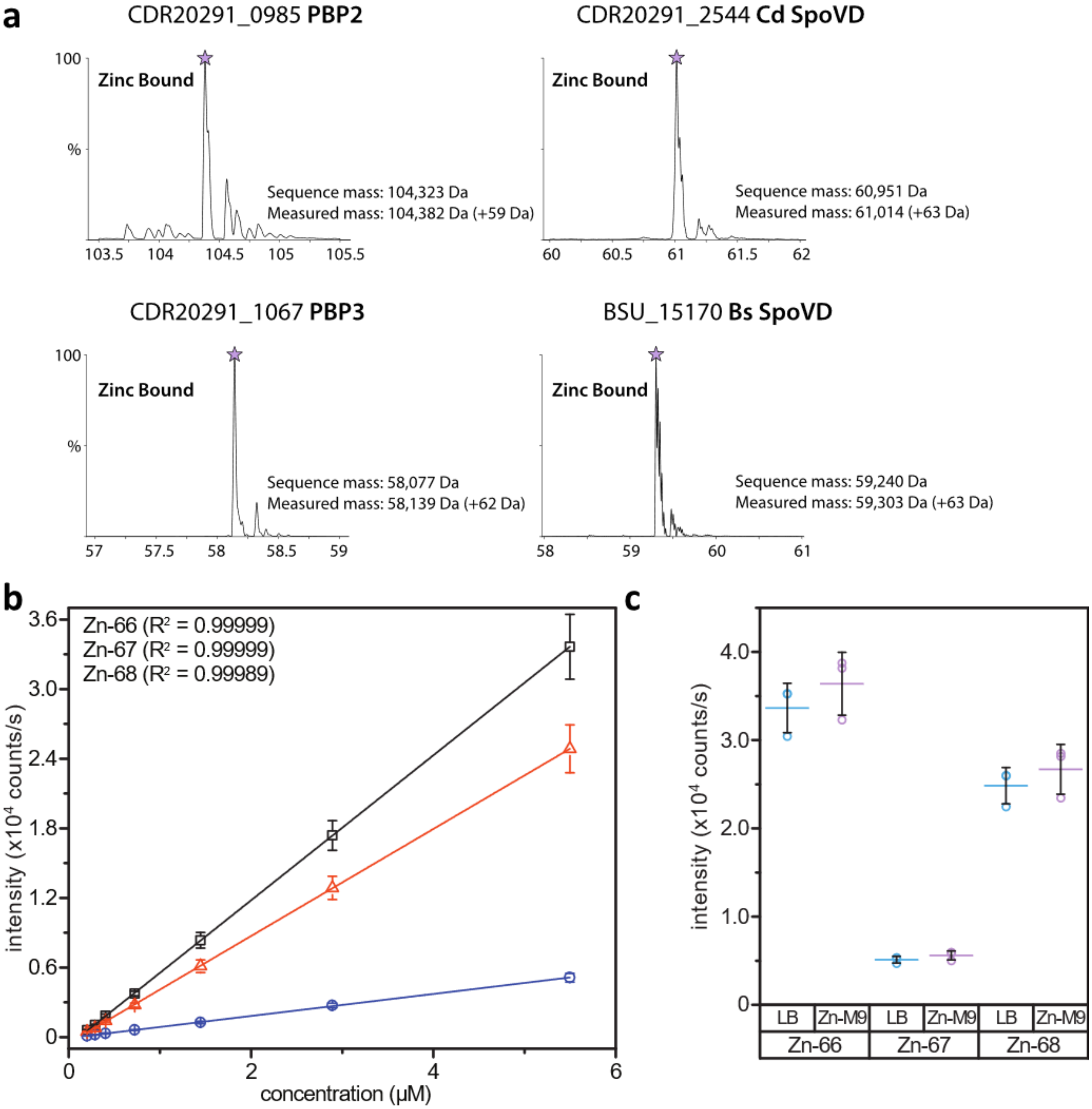


**Supplementary Figure 9| (a)** Native mass spectrometry shows *C. difficile* PBP2, SpoVD, PBP3, and *B. subtilis* SpoVD contain a mass peak corresponding to a $Zn^{2+}$-ion. **(b)** ICP-MS response for the three stable Zn isotopes (expressed as counts/s) exhibit a strong correlation to PBP3 concentration. For each concentration, error bars represent standard deviations of three technical measurement repeats. **(c)** Dot plot of the ICP-MS response of Zn-66, Zn-67 and Zn-68 for PBP3 expressed in rich media (LB), where the metal binding site may pick any ion (blue symbols), and PBP3 expressed in minimal media supplemented only with $ZnSO_4$ (pink symbols). Data recorded at 5.50 µM for PBP3 is shown. The values of three technical replicates (circles), the mean (horizontal lines) and the SD (error bars) are shown. Source data are provided as a Source Data file.

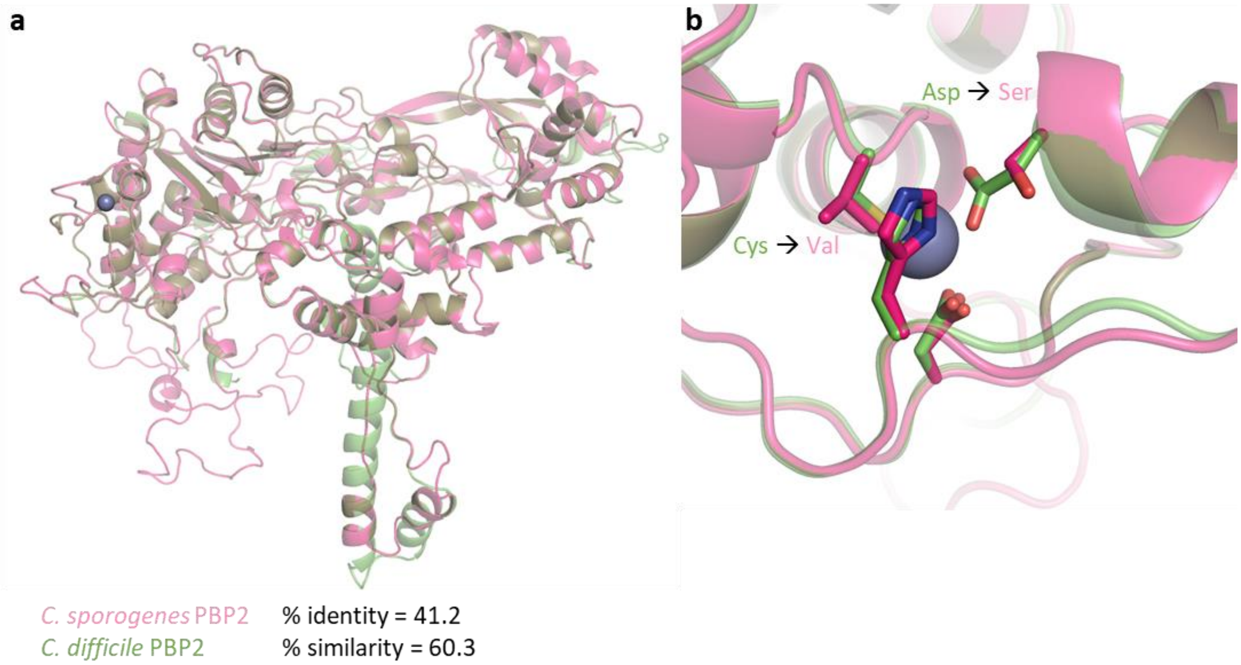


**Supplementary Figure 10| *C. sporogenes* PBP2 bears high similarity to *C. difficile* PBP2 but lacks a $Zn^{2+}$-binding motif. (a)** Structural superimposition of *C. difficile* PBP2 with homology model of *C. sporogenes* PBP2. *C. sporogenes* is remarkably similar in identity, but lacks a coiled-coil stretch and **(b)** residues capable of coordinating a $Zn^{2+}$ ion. Sequence similarity was defined by the BLOSUM62 matrix.

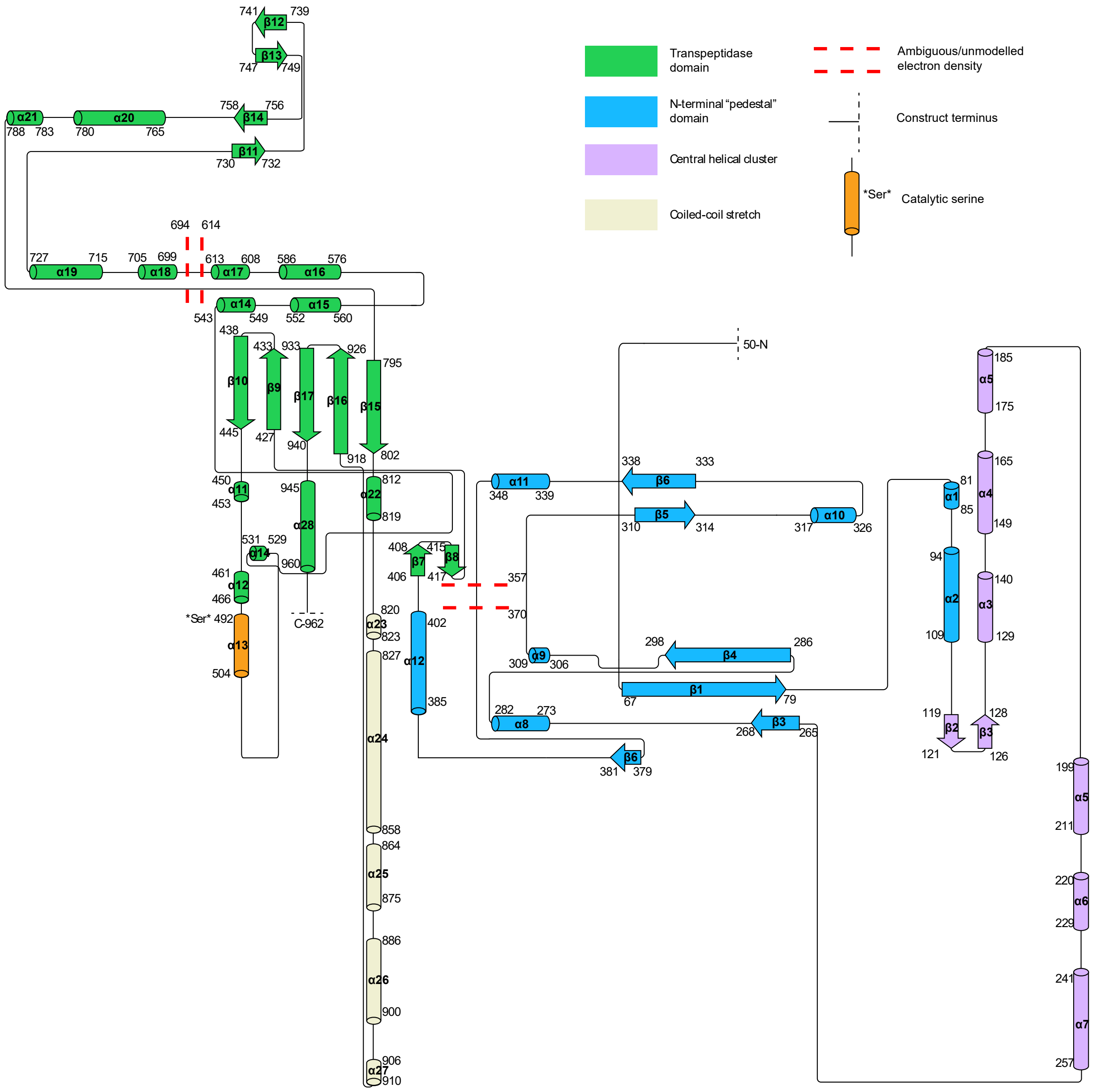


**Supplementary Figure 11| Topology Diagram of *C. difficile* PBP2**

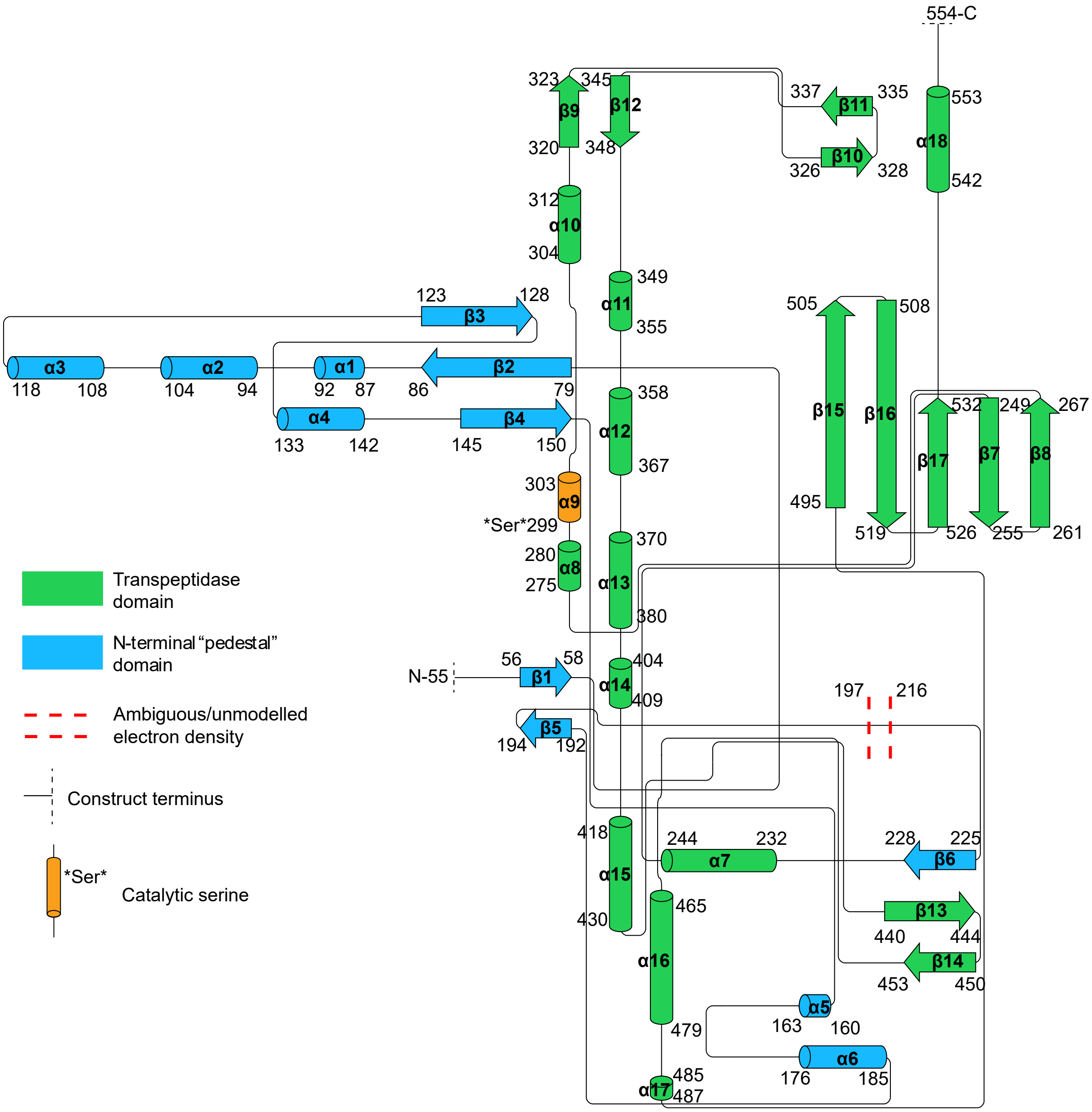

554-C
553
α18
542
323
345
β9
β12
320
348
337
335
β11
β10
326
328
312
α10
304
349
α11
355
123
128
β3
505
508
β15
β16
495
519
α3
α2
α1
β2
118
108
104
94
92
87
86
79
α4
β4
133
142
145
150
358
α12
367
532
249
267
β17
β7
β8
526
255
261
303
α9
*Ser*299
280
α8
275
370
α13
380
Transpeptidase domain
N-terminal "pedestal" domain
N-55
56
58
β1
404
α14
409
197
216
Ambiguous/unmodelled electron density
β5
194
192
Construct terminus
418
α15
430
244
232
α7
228
225
β6
*Ser*
Catalytic serine
465
α16
479
β13
440
444
β14
453
450
α5
163
160
α6
176
185
485
α17
487

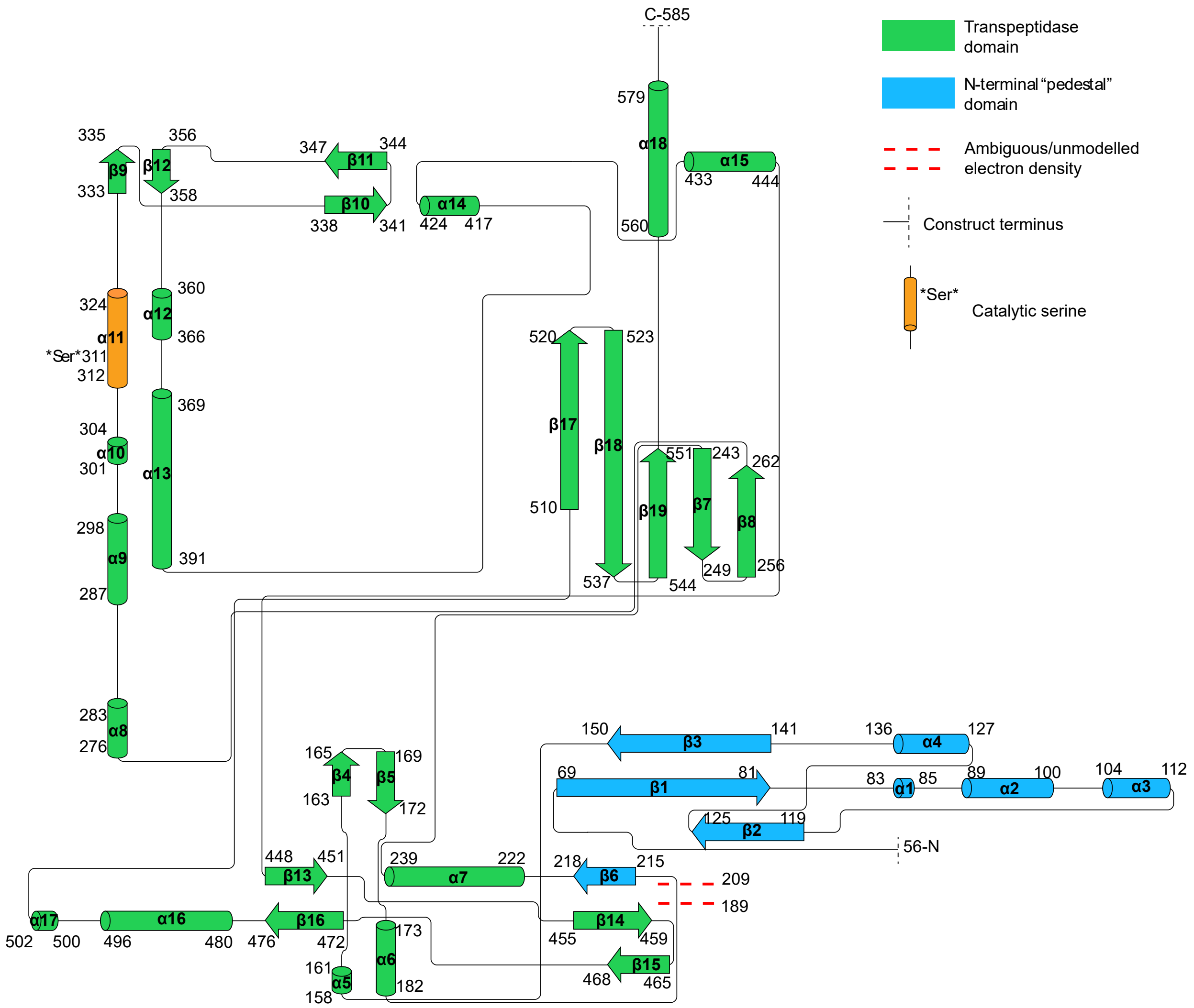


**Supplementary Figure 13| Topology Diagram of *C. difficile* SpoVD**

| Strain | RT | Clade | TPase PBPs # | CDR20291_0712 (PBP1) analog, % identity | CDR20291_0985 (PBP2) analog, % identity | CDR20291_1067 (PBP3) analog, % identity | CDR20291_2544 (SpoVD) analog, % identity | Fifth TPase PBP |
|---|---|---|---|---|---|---|---|---|
| R20291 | 027 | 2 | 4 | CDR20291_0712, 100% | CDR20291_0985, 100% | CDR20291_1067, 100% | CDR20291_2544, 100% | - |
| CD196 | 027 | 2 | 4 | >WP_004454690.1* | >WP_009899751.1, 99.9% | >WP_003438168.1, 100% | >WP_009893708.1, 100% | - |
| 630 | 012 | 1 | 4 | >WP_011860956.1, 99.4% | >WP_011861128.1, 99.8% | >WP_003438168.1, 100% | >WP_009893708.1, 100% | - |
| BR81 | 017 | 4 | 4 | >WP_003437513.1, 99.2% | >WP_003438055.1, 99.7% | >WP_003438168.1, 100% | >WP_009893708.1, 100% | - |
| M68 | 017 | 4 | 5 | >WP_021387666.1, 99.8% | >WP_022616428.1, 99.5% | >WP_021387575.1, 98.9% | >WP_009893708.1, 100% | >WP_021387575.1, 40.3 % B. subtilis PBP3 |
| QCD-63q42 | 001 | 1 | 5 | >WP_009895821.1, 99.1% | >WP_009896080.1, 99.6 % | >WP_009896136.1, 99.6 % | >WP_009893708.1, 100% | >WP_009895502.1, 41.3 % B. subtilis PBP3 |
| ATTC 9689 DSM 1296 | 001 | 1 | 5 | >WP_018112771.1, 96.2% | >WP_016729515.1, 99.6% | >WP_009896136.1, 99.6 % | >WP_009893708.1, 100% | >WP_009895502.1, 41.3 % B. subtilis PBP3 |
| NAP007 | 078 | 5 | 5 | >WP_003418435.1, 98.1% | >WP_003419055.1, 99.6% | >WP_003419244.1, 97.3 % | >WP_003416063.1, 99.5% | >WP_022615397.1, 41.6% B. subtilis PBP3 |
| NAP008 | 078 | 5 | 5 | >WP_003418435.1, 98.1% | >WP_003419055.1, 99.6% | >WP_003419244.1, 97.3 % | >WP_003416063.1, 99.5% | >WP_022615397.1, 41.6% B. subtilis PBP3 |
| DH/ NAP11/ 106/ST-46 | 106 | 1 | 4 | >WP_003437513.1, 99.2% | >WP_016729515.1, 99.6% | >WP_003438168.1, 100% | >WP_009893708.1, 100% | - |

**Supplementary Table 1| TPase PBPs amongst different C. difficile strains.** The strain, ribotype, # of TPase PBPs, and % similarity to R20291 PBPs analyzed in the study amongst notable ribotypes. *indicates incomplete accession.

| PDB ID | **7RCX** | **7RCW** | **7RCY** | **7RD0** | **7RCZ** |
|---|---|---|---|---|---|
| Gene (*R20291*) | *0985* | *0985* | *0985* | *1067* | *2544* |
| Protein | PBP2 | PBP2 | PBP2 | PBP3 | SpoVD |
| Inhibitor | Unbound | Ampicillin | Ceftobiprole | Unbound | Ampicillin |
| **Data Collection** | | | | | |
| Space Group | P 1 21 1 | P 1 21 1 | P 1 21 1 | C 2 2 21 | P 21 21 21 |
| Stoichiometry | 1mer | 1mer | 1mer | 1mer | Homo 2mer |
| Cell Dimensions | | | | | |
| a, b, c (Å) | 65.30, 122.46, 70.73 | 62.91, 200.19, 70.68 | 64.70, 196.39, 70.97 | 85.02 114.22, 154.63 | 76.64, 95.62, 176.77 |
| α, β, γ (°) | 90.00, 103.40, 90.00 | 90.00, 100.11, 90.00 | 90.00, 105.66, 90.00 | 90.00, 90.00, 90.00 | 90.00, 90.00, 90.00 |
| Resolution (Å) | 48.81 - 2.85 | 49.34 – 3.00 | 53.84 - 3.00 | 45.94 - 2.40 | 49.53 - 2.20 |
| | (3.00 - 2.85) | (3.16 – 3.00) | (3.11 - 3.00) | (2.49 - 2.40) | (2.25 - 2.20) |
| $R_{merge}$ | 0.067 (0.406) | 0.103 (0.580) | 0.067 (0.425) | 0.078 (0.916) | 0.095 (0.461) |
| <I>/σ<I> | 10.6 (2.1) | 9.2 (2.4) | 9.3 (2.0) | 8.3 (2.0) | 7.3 (2.5) |
| Completeness (%) | 92.2 (86.0) | 90.2 (86.1) | 83.9 (81.6) | 99.9 (99.9) | 94.6 (96.8) |
| Wilson B-factor (Å$^2$) | 51.70 | 43.70 | 63.75 | 50.00 | 32.41 |
| CC1/2 (%) | 99.4 (75.3) | 98.9 (77.4) | 98.1 (67.5) | 99.2 (74.3) | 98.5 (85.9) |
| Redundancy | 3.1 (2.9) | 3.6 (3.6) | 2.6 (2.6) | 5.7 (5.8) | 3.5 (3.4) |
| **Refinement** | | | | | |
| Resolution (Å) | 39.78 - 2.85 | 49.34 – 3.00 | 53.84 – 3.00 | 42.51 - 2.40 | 42.05 - 2.20 |
| | (2.95 - 2.85) | (3.11 - 3.00) | (3.16 - 3.00) | (2.49 - 2.40) | (2.28 - 2.20) |
| No. reflections/free | 23206 / 1135 | 30828 / 1505 | 28434 / 1483 | 29753 / 1528 | 62337 / 3188 |
| $R_{work}/R_{free}$ | 0.220 / 0.275 | 0.208 / 0.257 | 0.215 / 0.269 | 0.194 / 0.236 | 0.193 / 0.228 |
| Clashscore | 9.14 | 7.81 | 10.78 | 3.07 | 4.28 |
| No. Atoms | | | | | |
| Overall | 6377 | 6500 | 6527 | 3907 | 8389 |
| Protein | 6343 | 6430 | 6479 | 3823 | 7929 |
| Ligand/Ion | 22 | 42 | 37 | 25 | 141 |
| Water | 12 | 28 | 11 | 59 | 319 |
| B-Factors (Å$^2$) | | | | | |
| Overall | 74.52 | 69.55 | 85.20 | 63.47 | 44.39 |
| Protein | 74.52 | 69.72 | 85.28 | 63.53 | 44.09 |
| Ligand/Ion | 91.57 | 59.87 | 87.85 | 84.72 | 76.14 |
| Solvent | 40.05 | 43.65 | 42.62 | 50.24 | 37.79 |
| RMS Deviations | | | | | |
| Bond Lengths (Å) | 0.014 | 0.014 | 0.014 | 0.014 | 0.014 |
| Bond Angles (°) | 1.80 | 1.93 | 1.83 | 1.93 | 1.87 |
| Ramachandran Favored (%) | 91.89 | 89.34 | 90.88 | 94.56 | 96.91 |
| Ramachandran Allowed (%) | 7.99 | 10.66 | 9.00 | 4.60 | 2.89 |
| Ramachandran Outliers (%) | 0.12 | 0.00 | 0.12 | 0.84 | 0.20 |
| Rotameric Outliers (%) | 4.06 | 3.58 | 5.26 | 2.08 | 2.43 |

**Supplementary Table 2 | Table of crystallization statistics**. Values in parentheses correspond to highest-resolution shell. All datasets were each collected from a single crystal.

1 Pettersen, E. F. *et al.* UCSF Chimera--a visualization system for exploratory research and analysis. *Journal of Computational Chemistry* **25**, 1605-1612 (2004).
