# Supplementary material for: A unique class of Zn2+-binding serine-based PBPs underlies cephalosporin resistance and sporogenesis in Clostridioides difficile
Source: Nat Commun. 2022 Jul 28;13:4370. doi: 10.1038/s41467-022-32086-6 (PMC9334274; doi:10.1038/s41467-022-32086-6)
Supplement: Supplementary file 3 — Description of Additional Supplementary Files [file 41467_2022_32086_MOESM3_ESM.pdf]

Title: Supplementary Data 1
Description: Bacteria with PBPs containing Zn^{2+}-binding motifs.

Title: Supplementary Data 2
Description: PBP proteins for phylogenetic analysis. PBP proteins containing a Zn^{2+}-binding motif are shaded red, those without are shaded blue.
